# Overweight and obesity management strategies in survivors of paediatric acute lymphoblastic leukaemia: a systematic review protocol

**DOI:** 10.1136/bmjopen-2018-022530

**Published:** 2018-06-22

**Authors:** Salma Ladhani, Brianna Empringham, Kuan-Wen Wang, Carol Portwine, Laura Banfield, Russell J de Souza, Lehana Thabane, M Constantine Samaan

**Affiliations:** 1 Department of Pediatrics, McMaster University, Hamilton, Ontario, Canada; 2 Division of Pediatric Endocrinology, McMaster Children’s Hospital, Hamilton, Ontario, Canada; 3 Medical Sciences Graduate Program, McMaster University, Hamilton, Ontario, Canada; 4 Division of Pediatric Hematology/Oncology, McMaster Children’s Hospital, Hamilton, Ontario, Canada; 5 Health Sciences Library, McMaster University, Hamilton, Ontario, Canada; 6 Department of Health Research Methods, Evidence and Impact, McMaster University, Hamilton, Ontario, Canada; 7 Department of Anesthesia, McMaster University, Hamilton, Ontario, Canada; 8 Centre for Evaluation of Medicines, St. Joseph’s Health Care, Hamilton, Ontario, Canada; 9 Biostatistics Unit, St Joseph’s Healthcare, Hamilton, Ontario, Canada

**Keywords:** cancer survivorship, obesity, cancer aftereffects, lifestyle interventions, acute lymphoblastic leukemia

## Abstract

**Introduction:**

Acute lymphoblastic leukaemia is the most common paediatric cancer. Survivors of childhood acute lymphoblastic leukaemia (SALL) are at risk of obesity and related cardiometabolic diseases including type 2 diabetes, hypertension, stroke and cardiovascular events. Therefore, it is important to address obesity in this population as this may help mitigate future cardiometabolic comorbidities. In this systematic review, we aim to assess current treatment strategies including lifestyle interventions, pharmacotherapy and bariatric surgery to manage overweight and obesity in SALL.

**Methods and analysis:**

We will search the following databases for primary studies: CINAHL, SPORTDiscus, EMBASE, MEDLINE, PsycINFO, Cochrane Central Register of Controlled Trials, and Cochrane Database of Systematic Reviews. In addition, unpublished primary studies will be searched in ClinicalTrials.gov as well as conference proceedings, presentations, abstracts, editorials and ProQuest Dissertations and Theses A&I. Reviewers will perform title, abstract, and full-text screening as well as data abstraction and risk of bias assessment independently with a third reviewer to be consulted to resolve disagreements. Searches will be run and updated through May 1st, 2018. The overall quality of the evidence will be determined using the Grading of Recommendations, Assessment, Development, and Evaluation criteria for each outcome. A meta-analysis will be performed if two studies deploying similar interventions, populations, and design and outcomes are identified.

**Ethics and dissemination:**

As individual patient data will not be included, we do not require ethics approval. This review will be published in a peer-reviewed journal.

**PROSPERO registration number:**

CRD42016051031.

**Strengths and limitations of this study**

- This study includes a comprehensive search strategy across several databases to ensure the inclusion of representative studies.
- There is no limitation on language or the time of publication of papers included.
- We plan to use the Grading of Recommendations, Assessment, Development and Evaluation tool to analyse the overall quality of evidence.
- We expect high heterogeneity across studies, which may lead to challenges in performing a meta-analysis.

## Background

Childhood leukaemia is the most common paediatric cancer,^1^ with the most common subtype being acute lymphoblastic leukaemia (ALL). An estimated 2670 children were diagnosed with ALL in the USA in 2014, representing 26% of all childhood cancer diagnoses. There are close to 380 000 survivors of childhood cancer living in the USA, the majority of whom are over 20 years of age and are leukaemia survivors.^1^

Remarkable advances in the characterisation and treatment of ALL have improved survival rates, with up to 90% of children diagnosed with ALL surviving beyond 5 years today.^2 3^ However, survivors of childhood ALL (SALL) are at risk of diseases that influence their long-term outcomes.

One of the common morbidities seen in this population is obesity.^4 5^ It has been reported that 38% of SALL are overweight or obese, compared to 31% of the general paediatric population.^4^ When central obesity is associated with dysglycaemia, hypertension and dyslipidaemia it is referred to as the metabolic syndrome, which is a risk factor for cardiovascular disease and type 2 diabetes.^4 6^ The prevalence of the metabolic syndrome in SALL is slightly higher than that in the general population.^7^

Despite having only modestly higher obesity rates compared to non-cancer controls, SALL have a disproportionately increased risk of insulin resistance, type 2 diabetes, cardiovascular disease, stroke and hypertension at a relatively young age.^8 9^

Several risk factors drive the risk of overweight/obesity and adverse cardiometabolic outcomes in survivors, including treatment with cranial irradiation and corticosteroids,^4 10–13^ young age at diagnosis (0–4 years),^11 14–16^ being overweight or obese at diagnosis^15 16^ and female sex.^11 16–18^

Addressing obesity in SALL is imperative, for the development of obesity in children can lead to obesity in adulthood and this raises the risk of adverse cardiometabolic outcomes.^19–21^

Understanding the risk factors of overweight and obesity in SALL may enable the development of targeted interventions to improve long-term health outcomes by lowering the risk of obesity and its comorbidities. This systematic review aims to assess current interventions to manage overweight and obesity in SALL, and to evaluate their impact on adiposity and associated metabolic comorbidities.

### Research question

In SALL, are lifestyle interventions, pharmacotherapy or bariatric surgery effective in treating overweight and obesity?

## Objectives

1. To determine the effectiveness of existing interventions to manage overweight and obesity in SALL.
2. If feasible, to perform a meta-analysis of included studies and calculate a precise estimate of the effectiveness of management strategies in SALL.
3. Provide future research directions by identifying gaps in current evidence.

## Methods

The methodology for this protocol has been established and reported based on the Preferred Reporting Items for Systematic review and Meta-Analysis Protocols (PRISMA-P) guideline (see online supplementary file 1).^22 23^ When changes to the protocol are necessary, we will document the details for the rationale and the change in the reported systematic review.

10.1136/bmjopen-2018-022530.supp1 Supplementary data

### Patient and public involvement

Patients and the public were not involved in the design of this review protocol.

### Eligibility criteria

Eligible studies will include SALL diagnosed before the age of 18 years. All subtypes of ALL will be eligible including T and B cell leukaemias. Studies involving all interventions targeting overweight and obesity in this population will be included. If there are studies that include other types of childhood cancers, we will extract the data for the SALL population exclusively. If this information is not reported, we will contact the study Principal Investigators to obtain the data.

We will include interventions encompassing lifestyle, pharmacotherapy and bariatric surgery. Lifestyle interventions are defined as treatments that mainly involve exercise and dietary modifications. Pharmacotherapy involves the use of medications for the purpose of weight management. Bariatric surgery is defined as any surgical intervention with the primary goal of treating obesity. All current bariatric surgery procedures will be incorporated into this review including gastric bypass, biliopancreatic diversion with duodenal switch, sleeve gastrectomy and gastric banding.^24^

The eligible study designs include randomised controlled trials (RCTs), quasi RCTs, case–control studies, controlled or uncontrolled studies with before-and-after comparisons, and cross-sectional studies. Review articles, including systematic reviews, will be searched for relevant references if applicable to the research question. Conference proceedings, presentations, abstracts and editorials will be searched for relevant references as well. We will not restrict the language of publication to English. Studies published up to May 1st, 2018 will be included.

## Outcome measures

### Primary outcome

The primary outcome of this review involves comparing changes in body mass index z-score before and after the intervention.

### Secondary outcomes

The secondary outcomes will include changes in adiposity measures including waist circumference, hip circumference, waist-to-hip ratio, waist-to-height ratio and body fat percentage when reported. In addition, we will evaluate changes in insulin sensitivity by measuring the Homeostasis Model Assessment-Insulin resistance (HOMA-IR) or clamp studies, blood pressure (mmHg), non-alcoholic fatty liver disease status by liver ultrasound or elevated transaminases, dyslipidaemia (including cholesterol, triglycerides, high-density lipoprotein and low-density lipoprotein) and obstructive sleep apnoea based on sleep studies.

We will also document any reported adverse effects of the interventions. For lifestyle interventions, these include injuries and back pain.^25 26^ For pharmacotherapy, these encompass headaches and insomnia among others.^27^ For bariatric surgery, these include perioperative outcomes and surgical complications.^28^ Other adverse effects will be described if reported.

### Search strategy

When designing the search strategy, we will consult with a Senior Health Sciences Librarian with expertise in designing search strategies for systematic reviews. A sample MEDLINE search strategy is reported in online supplementary file 2. Searches will be conducted in CINAHL, SPORTDiscus, EMBASE, MEDLINE, PsycINFO, Cochrane Central Register of Controlled Trials, and Cochrane Database of Systematic Reviews. To identify grey literature, we will search ClinicalTrials.gov and ProQuest Dissertations and Theses A&I. Conference proceedings, presentations, abstracts, and editorials as well as references in reviews including systematic reviews will be examined for eligible articles.

10.1136/bmjopen-2018-022530.supp2 Supplementary data

### Data management

We will use EndNote X7^29^ to remove duplicates from the search results, and references will be exported to an excel file.

### Data screening

**Figure 1:**
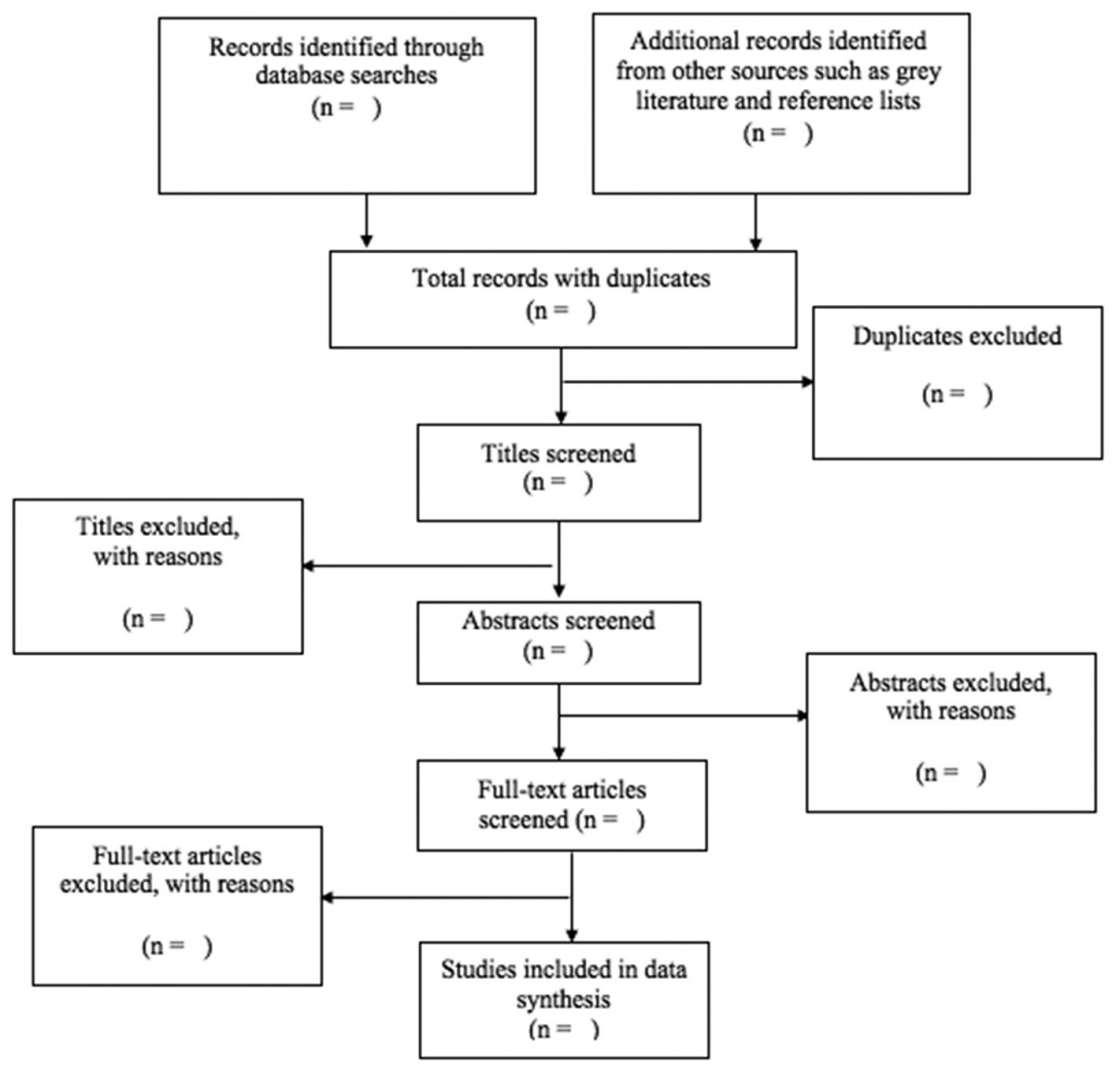
Flow diagram of article screening process.

We will perform initial screening by two teams, each composed of two reviewers independently for titles and abstracts eligibility. Differences in the included titles and abstracts will be discussed between the reviewers in each team. A third reviewer will address unresolved conflicts at each step of the review process. The reviewers will screen all abstracts, and if there is uncertainty of the eligibility of the abstracts, they will be included. For abstracts deemed eligible for inclusion, the full-text papers will be retrieved for full review. A flow diagram is included here and a populated one will be included in the full review to track the process (figure 1).

### Data abstraction

Pertinent data will be abstracted using a data abstraction form specifically designed for this project. This form will incorporate study details including authors, title, study date, country, study design, inclusion/exclusion criteria, journal of publication and funding details. It will also incorporate participant details including age, sex, ethnicity, age at ALL diagnosis and time from diagnosis to intervention if available.

Other data extracted include treatment types with radiotherapy field (cranial, craniospinal, total body irradiation), radiation type (fractionated vs non-fractionated) and radiation dose (Gy). We will also include chemotherapy protocols, name of medications, dose and duration of treatment. In addition, we will collect data on bone marrow transplantation, and medical and metabolic comorbidities will be abstracted.

The targeted obesity intervention details will be abstracted including study design, intervention details, duration and follow-up periods and any reported side effects.

Details of the statistics performed and results will be abstracted. In order to retrieve incomplete data, we will attempt to contact the Principal Investigators for each project. Outcomes will be summarised along with the overall conclusions and any confounding factors.

### Risk of bias and quality assessment

The risk of bias assessment tool created by the Cochrane Collaboration will be used to evaluate the risk of bias for RCTs.^30^ The studies will be rated as having a high, low or unclear risk of bias based on the validity of sequence generation, allocation concealment, blinding, data completeness, selective reporting and other sources of bias.

Non-randomised trials will be assessed using the Risk of Bias in Non-Randomised Studies—of Interventions (ROBINS-I) tool.^31^ This tool considers confounding variables and selection bias at the preintervention stage. The following confounding factors will be considered when using this tool: age at time of enrolment in the intervention, age at diagnosis, pubertal stage, sex, baseline body composition, treatments received for ALL, years since end of therapy and comorbidities such as metabolic syndrome and hormonal deficiencies. At the intervention stage, misclassification of intervention group will be evaluated. Lastly, deviation from intended interventions, missing data, standardised methods of outcome measures and selective reporting will be assessed at the postintervention stage.

In uncontrolled before and after studies, the risk of bias will be assessed using the University of Alberta Evidence-based Practice Center checklist.^32^ This checklist evaluates patient enrolment, incomplete data and standardised approach to outcomes to determine the risk of bias.

The overall quality of the body of evidence will be evaluated with the Grading of Recommendations, Assessment, Development and Evaluation (GRADE) approach.^33^ We will consider the quality of the evidence and the magnitude of the effect to determine the overall strength of the meta-analysis findings if it is feasible to do so. The determinants of quality include risk of bias, inconsistency, indirectness, imprecision and publication bias. The overall strength of evidence will be classified as high, moderate, low or very low for each outcome measure.

### Data analysis

We will include the summary and details of each relevant study in our analysis. If two or more studies of similar interventions, populations, study design, and outcomes are identified, a meta-analysis will be conducted. Categorical outcomes will be reported as odds ratios, and continuous variables will be reported as mean differences with 95% CI.

We will perform the meta-analysis with a random effects model if more than ten studies can be included using Review Manager software.^34^ We are expecting high heterogeneity across studies, and this random effects model is the preferred method in this case.^35^ Otherwise, we will present results from both random and fixed effects models.^36^

Heterogeneity across studies will be evaluated with inconsistency index (I^2^) and p values from Χ^2^ test for homogeneity, with I^2^ >75% and a p value of <0.10 representing considerable heterogeneity.^37^

If appropriate, we will perform subgroup meta-analysis by sex, as female SALL have been reported to have higher risk of obesity than male SALL and may respond to overweight and obesity management strategies differently.^11 16–18^ If possible, subgroup analyses will also be done based on treatment modalities, including those treated for a relapse or receiving a bone marrow transplantation. We will determine publication bias by creating a funnel plot if ten or more studies are included.^38^ We will use Egger’s test, performed with PASW V.18 statistical package,^39^ and visual inspection to determine the plot asymmetry.

Alternatively, we will estimate publication bias by considering the number of relevant conference abstracts without published articles. If a meta-analysis is not appropriate, a summary table with narrative description will be reported.

## Discussion

Improvements in treatments and supportive care have led to an increased survival in children diagnosed with ALL. However, comorbidities in SALL are high and affect the quality of life and long-term outcomes. The increased rates of obesity in this population represent a potential area of need for intervention to help improve cardiometabolic outcomes in SALL.

Around 50% of SALL have at least one chronic health condition compared to 37.8% of sibling controls.^40^ It is vitally important to understand if effective overweight and obesity interventions are available for SALL, as ALL treatment is associated with high cure rates. As survivors are forced to live longer with the burden of their cancer history, this study will summarise the current scientific understanding of interventions that target overweight and obesity in SALL, and will identify gaps in current research to guide future study design to improve outcomes.

## Ethics and dissemination

We will not include individual patient data in this review. Therefore, ethics approval is not required, and there are no further ethical and safety considerations. The dissemination plan for this review is to be published in a peer-reviewed journal.

## Supplementary Material

Reviewer comments

Author's manuscript
