# Supplementary material for: Overweight and obesity management strategies in survivors of paediatric acute lymphoblastic leukaemia: a systematic review protocol
Source: BMJ Open. 2018 Jun 22;8(6):e022530. doi: 10.1136/bmjopen-2018-022530 (PMC6020978; doi:10.1136/bmjopen-2018-022530)
Supplement: Supplementary data [file bmjopen-2018-022530supp002.pdf]

Supplementary File 2: Search Strategy for MEDLINE

1. exp Precursor Cell Lymphoblastic Leukemia-Lymphoma/
2. lymphoblastic leuk?emia*.mp.
3. lymphoblastic lymphoma*.mp.
4. lymphocytic leuk?emia*.mp.
5. lymphoid leuk?emia*.mp.
6. acute t-cell leuk?emia*.mp.
7. lymphatic leuk?emia*.mp.
8. t-lymphocytic leuk?emia*.mp.
9. precursor t cell lymphoblastic leuk?emia*.mp.
10. t cell leuk?emia*.mp.
11. t lymphocytic leuk?emia*.mp.
12. or/1-11
13. exp Overweight/
14. obes*.mp.
15. overweight.mp.
16. over weight.mp.
17. or/13-16
18. Life Change Events/
19. Life Style/
20. lifestyle*.mp.
21. life style*.mp.
22. exp Diet/

23. diet*.mp.

24. exp Nutrition Therapy/

25. nutrition*.mp.

26. behavio?r*.mp.

27. exp Exercise Therapy/

28. kinesiotherap*.mp.

29. physical* activ*.mp.

30. exp Exercise/

31. exercis*.mp.

32. ((strength or weight or resistance) adj1 train*).mp.

33. walk*.mp.

34. jog*.mp.

35. run*.mp.

36. swim*.mp.

37. exp Bariatrics/

38. bariatric*.mp.

39. gastrojejunostom*.mp.

40. gastric bypass*.mp.

41. stomach bypass*.mp.

42. jejunoileal bypass*.mp.

43. lipectom*.mp.

44. gastroplast*.mp.

45. stomach stapl*.mp.

46. exp Anti-Obesity Agents/

47. (drug or drugs).mp.

48. pharma*.mp.

49. Weight Reduction Programs/

50. ((weight reduc* or weight lo?s*) adj5 (strateg* or surger* or program* or system* or supplement*)).mp.

51. or/13-50

52. 12 and 17 and 51
