# Supplementary material for: Overweight and obesity management strategies in survivors of paediatric acute lymphoblastic leukaemia: a systematic review protocol
Source: BMJ Open. 2018 Jun 22;8(6):e022530. doi: 10.1136/bmjopen-2018-022530 (PMC6020978; doi:10.1136/bmjopen-2018-022530)
Supplement: Author's manuscript [file bmjopen-2018-022530.draft_revisions.pdf]

BMJ Open

BMJ Open is committed to open peer review. As part of this commitment we make the peer review history of every article we publish publicly available.

When an article is published we post the peer reviewers' comments and the authors' responses online. We also post the versions of the paper that were used during peer review. These are the versions that the peer review comments apply to.

The versions of the paper that follow are the versions that were submitted during the peer review process. They are not the versions of record or the final published versions. They should not be cited or distributed as the published version of this manuscript.

BMJ Open is an open access journal and the full, final, typeset and author-corrected version of record of the manuscript is available on our site with no access controls, subscription charges or pay-per-view fees (http://bmjopen.bmj.com).

If you have any questions on BMJ Open's open peer review process please email info.bmjopen@bmj.com

BMJ Open

Overweight and obesity management strategies in survivors of pediatric Acute Lymphoblastic Leukemia: A systematic review protocol

Journal:	*BMJ Open*
Manuscript ID	bmjopen-2018-022530
Article Type:	Protocol
Date Submitted by the Author:	22-Feb-2018

Complete List of Authors:	Ladhani, Salma; McMaster University Department of Pediatrics,<br>Empringham, Brianna; McMaster University, Department of Pediatrics; McMaster Children's Hospital, Division of Pediatric Endocrinology<br>Wang, Kuan-Wen; McMaster University, Department of Paediatrics, Division of Pediatric Endocrinology<br>Portwine, Carol; McMaster University, Department of Pediatrics; McMaster Children's Hospital, Division of Pediatric Hematology/Oncology<br>Banfield, Laura; McMaster University, Health Sciences Library<br>de Souza, Russell; McMaster University, Department of Clinical Epidemiology & Biostatistics<br>Thabane, Lehana ; McMaster University, Clinical Epidemiology & Biostatistics<br>Samaan, M. Constantine; McMaster University, Pediatrics

Keywords:	Cancer survivorship, Obesity, Cancer aftereffects, Lifestyle interventions, Acute Lymphoblastic Leukemia

SCHOLARONE™
Manuscripts

Overweight and obesity management strategies in survivors of pediatric Acute Lymphoblastic Leukemia: A systematic review protocol

Salma Ladhani[1,2*], Brianna Empringham[1,2*], Kuan-Wen Wang[1,2,3], Carol Portwine[1,4], Laura Banfield[5], Russell J. de Souza[3,6], Lehana Thabane[3,6-9], M. Constantine Samaan[1,2,3,6#]

*These authors contributed equally to this work

Salma Ladhani: salmaladhani@gmail.com

Brianna Empringham: brianna.empringham@medportal.ca

Kuan-Wen Wang: wangkw2@mcmaster.ca

Carol Portwine: portwc@mcmaster.ca

Laura Banfield: banfie@mcmaster.ca

Russell J. de Souza: rdesouz@mcmaster.ca

Lehana Thabane: thabanl@mcmaster.ca

M. Constantine Samaan: samaanc@mcmaster.ca

Authors' affiliations:

[1]Department of Pediatrics, McMaster University, Hamilton, Ontario, Canada

[2]Division of Pediatric Endocrinology, McMaster Children's Hospital, Hamilton, Ontario, Canada

[3]Medical Sciences Graduate Program, McMaster University, Hamilton, Ontario, Canada

[4]Division of Pediatric Hematology/Oncology, McMaster Children's Hospital, Hamilton, Ontario, Canada

[5]Health Sciences Library, McMaster University, Hamilton, Ontario, Canada

For peer review only

[6]Department of Health Research Methods, Evidence and Impact, McMaster University, Hamilton, Ontario, Canada
[7]Department of Anesthesia, McMaster University, Hamilton, Ontario, Canada
[8]Centre for Evaluation of Medicines, St. Joseph's Health Care, Hamilton, Ontario, Canada
[9]Biostatistics Unit, St Joseph's Healthcare, Hamilton, Ontario, Canada

# Corresponding Author:
Dr. M. Constantine Samaan
Department of Pediatrics,
McMaster University,
Division of Pediatric Endocrinology
McMaster Children's Hospital,
1280 Main Street West, HSC-3A57,
Hamilton, Ontario L8S 4K1
Tel: 001-905-521-2100, ext. 75926,
Fax: 001-905-3087548
E-mail:samaanc@mcmaster.ca

Word count: 1980

For peer review only

For peer review only - http://bmjopen.bmj.com/site/about/guidelines.xhtml

Abstract

Introduction: Acute lymphoblastic leukemia is the most frequent pediatric cancer. Survivors of childhood acute lymphoblastic leukemia (SALL) are at risk of obesity and related cardiometabolic diseases including type 2 diabetes, hypertension, stroke and cardiovascular events. Therefore, it is important to address obesity in this population as this may help mitigate future cardiometabolic comorbidities. In this systematic review, our goal is to verify the effectiveness of current treatment strategies including lifestyle interventions, pharmacotherapy and bariatric surgery to manage overweight and obesity in SALL.

Methods and analysis: We will search databases including CINAHL, SPORTDiscus, EMBASE, MEDLINE, PsycINFO, Cochrane Central Register of Controlled Trials, and Cochrane Database of Systematic Review for relevant studies. In addition, unpublished primary studies will be searched in ClinicalTrials.gov and ProQuest Dissertations and Theses A&I. Reviewers will perform title, abstract, full-text screening, data abstraction, and risk of bias assessment independently with a third reviewer to be consulted to resolve disagreements. Searches will be run and updated through August 31st, 2017. The overall quality of evidence will be determined using the Grading of Recommendations, Assessment, Development, and Evaluation (GRADE) criteria for each outcome. Meta-analysis will be done if two studies using similar interventions with similar population and study design are identified.

Ethics and dissemination: As individual patient data will not be included, we do not require ethics approval. This review will be published in a peer-reviewed journal.

Systematic review registration: PROSPERO CRD42016051031

For peer review only

Keywords: Acute lymphoblastic leukemia, pediatrics, survivorship, obesity, systematic review, lifestyle, pharmacotherapy, bariatric surgery

Strengths and Limitations

- This study includes a comprehensive search strategy across several databases to ensure the inclusion of representative studies.
- There is no limitation on language or the time of publication of papers included.
- We plan to use the Grading of Recommendations, Assessment, Development, and Evaluation (GRADE) tool to analyse the overall quality of evidence, which provides a high-quality overview of the strength of the evidence.
- We expect high heterogeneity across studies, which may place challenges on performing a quantitative analysis of the evidence.

For peer review only

BACKGROUND

Childhood leukemia is the most common pediatric cancer,[1] with the most common subtype being acute lymphoblastic leukemia (ALL). An estimated 2670 children were diagnosed with ALL in the USA in 2014, representing 26% of all childhood cancer diagnoses.[1] There are close to 380,000 survivors of childhood cancer living in the USA, the majority of whom are over twenty years of age, and are leukemia survivors.[1]

Remarkable advances in the characterization and treatment of ALL have improved survival rates, with nearly 90% of children diagnosed with ALL surviving beyond five years today.[2 3] However, survivors of childhood ALL (SALL) are at risk of diseases that influence their long-term outcomes.

One of the common morbidities seen in this population is obesity.[4 5] It has been reported that the 38% of SALL are overweight or obese, compared to 31% of the general pediatric population.[4] Central obesity when associated with dysglycemia, hypertension and dyslipidemia is referred to as the metabolic syndrome, which is a risk factor for cardiovascular disease and type 2 diabetes.[4 6] The prevalence of metabolic syndrome in SALL is slightly higher than that in the general population.[7 8]

Despite having only modestly higher obesity rates compared to non-cancer controls, SALL have a disproportionately increased risk of insulin resistance and type 2 diabetes, cardiovascular disease, stroke, and hypertension at a relatively young age.[8-11]

Several risk factors drive the overweight/obesity and adverse cardiometabolic outcomes in survivors, including treatment with cranial irradiation and corticosteroids,[4 12-15] young age at diagnosis (0-4 years),[13 16-18] being overweight or obese at diagnosis,[17 18] and female sex.[13 18-20]

For peer review only - http://bmjopen.bmj.com/site/about/guidelines.xhtml

Addressing obesity in SALL is imperative, for the development of obesity in children can lead to obesity in adulthood and this raises the probability of adverse cardiometabolic outcomes.[21-23]

Understanding the risk factors of overweight and obesity in SALL will enable the development of targeted interventions, and this may improve health outcomes by lowering the risk of obesity and its comorbidities.

This systematic review aims to assess current interventions to manage overweight and obesity in SALL, and to evaluate their impact on adiposity and associated metabolic comorbidities.

RESEARCH QUESTION: In SALL, are lifestyle interventions, pharmacotherapy or bariatric surgery effective in treating overweight and obesity?

OBJECTIVES

1) To determine the effectiveness of existing interventions to manage overweight and obesity in SALL.
2) If feasible, to perform a meta-analysis of included studies and calculate a precise estimate of the effectiveness management strategies in SALL
3) Offer future research directions by identifying knowledge gaps

METHODS

The methodology for this protocol will be reported based on the Preferred Reporting Items for Systematic Review and Meta-Analysis Protocols (PRISMA-P) guideline (Supplementary File 1).[24 25] When changes to the protocol are necessary, we will document the details for the rationale and the changes made in the systematic review.

For peer review only - http://bmjopen.bmj.com/site/about/guidelines.xhtml

Eligibility criteria

The studies sought will include SALL diagnosed before the age of 18 years. All subtypes of ALL will be eligible including T and B cell leukemias. Studies that involve all interventions targeting overweight and obesity in this population will be included. If there are studies that include other types of childhood cancers, we will check for subgroup analysis by cancer type and extract the data for the SALL population exclusively. If this information is not reported, we will contact the study Principal Investigators to obtain the data. These studies will be included in the meta-analysis.

We will include interventions encompassing lifestyle, pharmacotherapy and bariatric surgery. Lifestyle interventions are defined as treatments that mainly involve exercise and dietary modifications. Pharmacotherapy involves the use of medications for the purpose of weight management. Bariatric surgery is defined as any surgical intervention with the primary goal of treating obesity. All current bariatric surgery procedures will be incorporated into this review including gastric bypass, biliopancreatic diversion with duodenal switch, sleeve gastrectomy, and gastric banding.[26]

The eligible study designs include randomized controlled trials (RCTs), quasi RCTs, case-control studies, controlled or uncontrolled studies with before-and-after comparisons, and cross-sectional studies.[27] Review articles, including systematic reviews, will be searched for relevant references if applicable to the research question. Conference proceedings, presentations, abstracts and editorials will be searched for relevant references as well. We will not restrict the language of publication. Studies published up to August 31st, 2017 will be included, and updated searches will be completed closer to submission to ensure the inclusion of new papers prior to the completion of this review.

For peer review only

Outcome measures

Primary outcome

The primary outcome of this review involves comparing changes in body mass index (BMI) z-score before and after the intervention.

Secondary outcomes

The secondary outcomes will include changes in adiposity measures including BMI, BMI percentile, waist circumference, hip circumference, waist-to-hip ratio, waist-to-height ratio, and body fat percentage when reported. In addition, we will evaluate changes in insulin sensitivity through glucose tolerance tests, blood pressure, non-alcoholic fatty liver disease status, dyslipidemia (including cholesterol, triglycerides, high-density lipoprotein, and low-density lipoprotein), and obstructive sleep apnea.

We will also document any reported adverse effects of the interventions. For lifestyle interventions, these include injuries, back pain, and angina.[28 29] For example, for pharmacotherapy these encompass headaches and insomnia among others.[30] For bariatric surgery, these include perioperative outcomes and surgical complications.[31] Other adverse effects will be described if reported.

Search Strategy

When designing the search strategy, we will consult a Senior Health Sciences Librarian with expertise in designing search strategies for systematic reviews. A sample MEDLINE search strategy is reported in Supplementary File 2. Searches will be conducted in CINAHL, SPORTDiscus, EMBASE, MEDLINE, PsycINFO, Cochrane Central Register of Controlled Trials, and Cochrane Database of Systematic Review. To identify grey literature, we will search ClinicalTrials.gov and ProQuest Dissertations and Theses A&I.

For peer review only - http://bmjopen.bmj.com/site/about/guidelines.xhtml

Data Management

We will use EndNote X7[32] to remove duplications from the search results, and references will be exported to an excel file.

Data Screening

We will remove duplicate references, and perform initial screening by two teams, each composed of two reviewers independently for titles and abstracts. Differences in the included titles and abstracts will be discussed between the two reviewers in each team. A third reviewer will address unresolved conflicts at each step of the review process. The reviewers will screen all abstracts, and if there is uncertainty in the eligibility of the abstracts, they will be included. For all abstracts deemed eligible for inclusion, the full-text papers will be retrieved and will undergo a full review by the two reviewers in each team independently. A flow diagram is included to demonstrate the tracking of the review process (Figure 1).

Data Abstraction

Pertinent data to the study question will be abstracted using a data abstraction form specifically designed for this project. This form incorporates study details including authors, title, study date, country, study design, inclusion/exclusion criteria, journal of publication, and funding details. It also incorporates participant details including age, sex, ethnicity, age at ALL diagnosis, and time from diagnosis to intervention if available.

Other data extracted include treatment types with radiotherapy field (cranial, craniospinal, total body irradiation), radiation type (fractionated versus non-fractionated) and radiation dose. We will also include chemotherapy protocols, the name of medications, dose, and duration of treatment. In addition, medical as well as metabolic comorbidities will be abstracted.

The targeted obesity intervention details will be abstracted including study design,

For peer review only - http://bmjopen.bmj.com/site/about/guidelines.xhtml

intervention details, duration and follow-up periods, and any reported side effects. Details of the statistics performed and results will be abstracted. In order to retrieve incomplete data, we will attempt to contact the Principal Investigators of the published work.

Risk of Bias & Quality Assessment

The Risk of Bias Assessment Tool created by the Cochrane Collaboration will be used to evaluate the risk of bias for RCTs.[33] The studies will be rated as having a high, low or unclear risk of bias based on the validity of sequence generation, allocation concealment, blinding, data completeness, selective reporting and other sources of bias.

Non-randomized trials, including cohort studies, will be assessed using the Risk of Bias in Non-Randomized Studies- of Interventions (ROBINS-I) tool.[34] This tool considers confounding variables and selection bias at the pre-intervention stage. The following confounding factors will be considered when using this tool: Age at time of enrolment in the intervention, age at diagnosis, pubertal stage, sex, baseline body composition, treatments received for ALL, years since end of therapy, and comorbidities such as metabolic syndrome and hormonal deficiencies. At the intervention stage, misclassification of intervention group will be evaluated. Lastly, deviation from intended interventions, missing data, standardized methods of outcome measures, and selective reporting will be assessed at the post-intervention stage.

In uncontrolled before and after studies, the risk of bias will be assessed using the University of Alberta Evidence-based Practice Center (UAEPC) checklist.[35] This checklist looks at patient enrollment, incomplete data and standardized approach to outcomes to determine the risk of bias.

The overall quality of the body of evidence will be evaluated with the Grading of Recommendations, Assessment, Development, and Evaluation (GRADE) approach.[36] We will

For peer review only

consider the quality of the evidence and the magnitude of the effect to determine the overall strength of the meta-analysis findings if it is feasible to do so. The determinants of quality include risk of bias, consistency of results across studies, indirectness/heterogeneity, imprecision and publication bias. The overall strength of evidence will be classified as high, moderate, low or very low for each outcome measure.

Data Analysis

For peer review only

We will include the summary and details of each relevant study in our analysis. If two studies on similar interventions with similar population and study design are identified, a meta-analysis will be conducted. Otherwise, a narrative summary with a detailed table of findings will be presented. Categorical outcomes will be conveyed as odds ratios, and continuous variables will be reported as mean differences with 95% confidence interval.

We will perform a meta-analysis with a random effects model if more than ten studies can be included for meta-analysis using Review Manager software.[37] We are expecting high heterogeneity across studies, and this random effects model is preferred in this case.[38] Otherwise, we will present results from both random and fixed effects models.[39]

Heterogeneity across studies will be evaluated with inconsistency index (I^2) and p-values from chi-square test for homogeneity, with I^2 >75% and a p-value of <0.10 representing considerable heterogeneity.[40]

If appropriate, we will perform subgroup meta-analysis by sex, as female SALL has been reported to have higher risk of obesity than male SALL and may respond to overweight and obesity management strategies differently.[13 18-20] If possible, subgroup analysis will also be done based on treatment modalities, including those who have been treated for a relapse and those receiving bone marrow transplantation. We will determine publication bias by creating a funnel

For peer review only - http://bmjopen.bmj.com/site/about/guidelines.xhtml

plot if ten or more studies are included.[41] We will use Egger's test, performed with PASW version 18 statistical package,[42] and visual inspection to determine the plot asymmetry. Alternatively, we will estimate publication bias by considering the number of relevant conference abstracts without published articles.[43] If a meta-analysis is not appropriate, a summary table with narrative description will be reported.

DISCUSSION

Improvements in treatments and supportive care have led to an increased survival in children diagnosed with ALL. However, comorbidities in SALL are high and affect quality of life and long-term outcomes. The increased rates of obesity in this population represent a potential area of need for intervention to help improve cardiometabolic outcomes in SALL. Around 50% of SALL have at least one chronic health issue, compared to 37.8% of siblings.[44] As this population gets older, survivors are forced to live longer with the burden of their cancer history. This study will summarize the current scientific understanding of interventions that target overweight and obesity in SALL, and will identify gaps in current research, and guide future study design to improve outcomes.

Ethics and dissemination: We will not include individual patient data in this review. Therefore, ethics approval is not required, and there are no further ethical and safety considerations. This review is to be published in a peer-reviewed journal.

For peer review only

ABBREVIATIONS

ALL: Acute Lymphoblastic Leukemia; SALL: Survivors of childhood Acute Lymphoblastic Leukemia; PRISMA-P: Preferred Reporting Items for Systematic Review and Meta-Analysis-Protocols; RCT: Randomized Control Trial; BMI: Body Mass Index; MEDLINE: Medical Literature Analysis and Retrieval System; EMBASE: Excerpta Medica Database; CINAHL: Cumulative Index to Nursing and Allied Health Literature; GRADE: Grading of Recommendations, Assessment, Development and Evaluation; ROBINS-I: Risk of Bias in Non-Randomized Studies – of Interventions; UAEPC: University of Alberta Evidence-based Practice Center.

For peer review only

DECLARATIONS

Ethics approval and consent to participate: Not applicable

Consent for publication: Granted

Availability of data and materials: Not applicable

Funding: MCS was funded by the Pediatric Oncology Group of Ontario (POGO) Research Unit, and the Hamilton Health Sciences and Foundation, KWW was funded by the Ontario Graduate Scholarship Program, and the Canadian Institutes of Health Research (CIHR) Canada Graduate Scholarship-Masters. Design of the systematic review is independent of the funding agencies.

Competing interests: Outside of the submitted work, RJdS received personal fees from Canadian Institutes for Health Research/Health Canada and World Health Organization. He also received grants from Dietitians of Canada and Canadian Institutes for Health Research. Lastly, he reports non-financial support from World Health Organization. The other authors declared no competing interests.

Authors' contributions: MCS is the guarantor. Research question was defined by SL, BE, CP, RJdS, LT and MCS. SL, BE, KWW, CP, LB, RJdS, LT and MCS contributed to the development of search strategy and determination of the eligibility criteria. SL, BE, LB, KWW and MCS designed the data abstraction form. RJdS and LT provided methodological support for this review. SL, BE, KWW, CP and MCS drafted the manuscript, and the final version was reviewed and approved by all authors.

Acknowledgements: We would like to thank Ms. E. Danielle Sims for assistance in addressing the editorial comments.

For peer review only

References

1. Ward E, DeSantis C, Robbins A, et al. Childhood and adolescent cancer statistics, 2014. *CA: A Cancer Journal for Clinicians* 2014;64(2):83-103. doi: 10.3322/caac.21219
2. Pui CH, Evans WE. Treatment of acute lymphoblastic leukemia. *N Engl J Med* 2006;354(2):166-78. doi: 10.1056/NEJMra052603 [published Online First: 2006/01/13]
3. Pulte D, Gondos A, Brenner H. Trends in 5- and 10-year Survival After Diagnosis with Childhood Hematologic Malignancies in the United States, 1990–2004. *JNCI: Journal of the National Cancer Institute* 2008;100(18):1301-09. doi: 10.1093/jnci/djn276
4. Chow EJ, Pihoker C, Hunt K, et al. Obesity and hypertension among children after treatment for acute lymphoblastic leukemia. *Cancer* 2007;110(10):2313-20. doi: 10.1002/cncr.23050 [published Online First: 2007/09/28]
5. Withycombe JS, Smith LM, Meza JL, et al. Weight change during childhood acute lymphoblastic leukemia induction therapy predicts obesity: a report from the Children's Oncology Group. *Pediatr Blood Cancer* 2015;62(3):434-9. doi: 10.1002/pbc.25316 [published Online First: 2014/11/20]
6. Expert Panel on Detection, Evaluation, and Treatment of High Blood Cholesterol in Adults. Executive Summary of The Third Report of The National Cholesterol Education Program (NCEP) Expert Panel on Detection, Evaluation, And Treatment of High Blood Cholesterol In Adults (Adult Treatment Panel III). *JAMA* 2001;285(19):2486-97. [published Online First: 2001/05/23]
7. Kourti M, Tragiannidis A, Makedou A, et al. Metabolic syndrome in children and adolescents with acute lymphoblastic leukemia after the completion of chemotherapy. *Journal of pediatric hematology/oncology* 2005;27(9):499-501. [published Online First: 2005/09/29]
8. Follin C, Gabery S, Petresen Æ, et al. High metabolic risk is associated with small hypothalamic volume in acute lymphoblastic leukemia survivors 34 years after cranial radiotherapy. *Endocrine Abstracts* 2015;37 EP660 doi: 10.1530/endoabs.37.EP660
9. Nottage KA, Ness KK, Li C, et al. Metabolic syndrome and cardiovascular risk among long-term survivors of acute lymphoblastic leukaemia - From the St. Jude Lifetime Cohort. *British journal of haematology* 2014;165(3):364-74. doi: 10.1111/bjh.12754 [published Online First: 2014/01/29]
10. Oeffinger KC. Are survivors of acute lymphoblastic leukemia (ALL) at increased risk of cardiovascular disease? *Pediatric Blood & Cancer* 2008;50(S2):462-67. doi: 10.1002/pbc.21410
11. Oeffinger KC, Adams-Huet B, Victor RG, et al. Insulin Resistance and Risk Factors for Cardiovascular Disease in Young Adult Survivors of Childhood Acute Lymphoblastic Leukemia. *Journal of Clinical Oncology* 2009;27(22):3698-704. doi: doi:10.1200/JCO.2008.19.7251
12. Garmey EG, Liu Q, Sklar CA, et al. Longitudinal changes in obesity and body mass index among adult survivors of childhood acute lymphoblastic leukemia: a report from the Childhood Cancer Survivor Study. *J Clin Oncol* 2008;26(28):4639-45. doi: 10.1200/jco.2008.16.3527 [published Online First: 2008/10/01]
13. Oeffinger KC, Mertens AC, Sklar CA, et al. Obesity in Adult Survivors of Childhood Acute Lymphoblastic Leukemia: A Report from the Childhood Cancer Survivor Study. *Journal of Clinical Oncology* 2003;21(7):1359-65. doi: doi:10.1200/JCO.2003.06.131
14. Sklar CA, Mertens AC, Walter A, et al. Changes in body mass index and prevalence of overweight in survivors of childhood acute lymphoblastic leukemia: role of cranial irradiation. *Medical and pediatric oncology* 2000;35(2):91-5. [published Online First: 2000/08/05]
15. van Beek RD, de Muinck Keizer-Schrama SM, Hakvoort-Cammel FG, et al. No difference between prednisolone and dexamethasone treatment in bone mineral density and growth in long term

For peer review only

survivors of childhood acute lymphoblastic leukemia. *Pediatr Blood Cancer* 2006;46(1):88-93. doi: 10.1002/pbc.20437 [published Online First: 2005/06/01]

16. Dalton VK, Rue M, Silverman LB, et al. Height and weight in children treated for acute lymphoblastic leukemia: relationship to CNS treatment. *J Clin Oncol* 2003;21(15):2953-60. doi: 10.1200/jco.2003.03.068 [published Online First: 2003/07/30]
17. Razzouk BI, Rose SR, Hongeng S, et al. Obesity in survivors of childhood acute lymphoblastic leukemia and lymphoma. *J Clin Oncol* 2007;25(10):1183-9. doi: 10.1200/jco.2006.07.8709 [published Online First: 2007/04/03]
18. Withycombe JS, Post-White JE, Meza JL, et al. Weight Patterns in Children With Higher Risk ALL: A Report From the Children's Oncology Group (COG) for CCG 1961. *Pediatric blood & cancer* 2009;53(7):1249-54. doi: 10.1002/pbc.22237
19. Kohler JA, Moon RJ, Wright S, et al. Increased adiposity and altered adipocyte function in female survivors of childhood acute lymphoblastic leukaemia treated without cranial radiation. *Horm Res Paediatr* 2011;75(6):433-40. doi: 10.1159/000324412 [published Online First: 2011/04/06]
20. Odame I, Reilly JJ, Gibson BE, et al. Patterns of obesity in boys and girls after treatment for acute lymphoblastic leukaemia. *Archives of Disease in Childhood* 1994;71(2):147-49.
21. Bray GA. Predicting obesity in adults from childhood and adolescent weight. *Am J Clin Nutr* 2002;76(3):497-8.
22. Guo SS, Wu W, Chumlea WC, et al. Predicting overweight and obesity in adulthood from body mass index values in childhood and adolescence. *Am J Clin Nutr* 2002;76(3):653-8.
23. Simmonds M, Llewellyn A, Owen CG, et al. Predicting adult obesity from childhood obesity: a systematic review and meta-analysis. *Obes Rev* 2016;17(2):95-107. doi: 10.1111/obr.12334
24. Moher D, Shamseer L, Clarke M, et al. Preferred reporting items for systematic review and meta-analysis protocols (PRISMA-P) 2015 statement. *Syst Rev* 2015;4:1. doi: 10.1186/2046-4053-4-1 [published Online First: 2015/01/03]
25. Moher D, Stewart L, Shekelle P. Implementing PRISMA-P: recommendations for prospective authors. *Systematic Reviews* 2016;5(1):15. doi: 10.1186/s13643-016-0191-y
26. Buchwald H, Oien DM. Metabolic/bariatric surgery Worldwide 2008. *Obes Surg* 2009;19(12):1605-11. doi: 10.1007/s11695-009-0014-5
27. Reeves BC, Deeks JJ, Higgins JPT, et al. Chapter 13: Including non-randomized studies. In: Higgins JPT, Green S, eds. Cochrane handbook for systematic reviews of intervneions version 510 [updated March 2011]: The Cochrane Collaboration, 2011.
28. Ried-Larsen M, Christensen R, Hansen KB, et al. Head-to-head comparison of intensive lifestyle intervention (U-TURN) versus conventional multifactorial care in patients with type 2 diabetes: protocol and rationale for an assessor-blinded, parallel group and randomised trial. *BMJ Open* 2015;5(12) doi: 10.1136/bmjopen-2015-009764
29. Yang Z, Scott CA, Mao C, et al. Resistance Exercise Versus Aerobic Exercise for Type 2 Diabetes: A Systematic Review and Meta-Analysis. *Sports Medicine* 2014;44(4):487-99. doi: 10.1007/s40279-013-0128-8
30. Kang JG, Park CY. Anti-Obesity Drugs: A Review about Their Effects and Safety. *Diabetes Metab J* 2012;36(1):13-25. doi: 10.4093/dmj.2012.36.1.13
31. Hopkins JC, Howes N, Chalmers K, et al. Outcome reporting in bariatric surgery: an in-depth analysis to inform the development of a core outcome set, the BARIACT Study. *Obesity Reviews* 2015;16(1):88-106. doi: 10.1111/obr.12240
32. EndNote [Computer program]. Version 7.7.1 [program]: Clarivate Analytics, 2016.
33. Higgins JPT, Altman DG, Sterne JAC. (editors). Chapter 8: Assessing risk of bias in included studies. In: Higgins JPT, Green S, eds. Cochrane handbook for systematic review of interventions Version 510 [updated March 2011]: The Cochrane Collaboration, 2011.

For peer review only

34. Sterne JA, Hernan MA, Reeves BC, et al. ROBINS-I: a tool for assessing risk of bias in non-randomised studies of interventions. *BMJ* 2016;355:i4919. doi: 10.1136/bmj.i4919 [published Online First: 2016/10/14]
35. Seida JC, Schouten JR, Mousavi SS, et al. 2. Methods. Comparative effectiveness of nonoperative and operative treatment for rotator cuff tears Rockville (MD): Agency for Healthcare Research and Quality (US), 2010.
36. Atkins D, Best D, Briss PA, et al. Grading quality of evidence and strength of recommendations. *BMJ* 2004;328(7454):1490. doi: 10.1136/bmj.328.7454.1490
37. Review Manager (RevMan) [Computer program]. Version 5.3. [program]. Copenhagen: The Nordic Cochrane Centre, The Cochrane Collaboration, 2014.
38. Villar J, Mackey ME, Carroli G, et al. Meta-analyses in systematic reviews of randomized controlled trials in perinatal medicine: comparison of fixed and random effects models. *Stat Med* 2001;20(23):3635-47.
39. Borenstein M, Hedges LV, Higgins JP, et al. A basic introduction to fixed-effect and random-effects models for meta-analysis. *Research synthesis methods* 2010;1(2):97-111. doi: 10.1002/jrsm.12 [published Online First: 2010/04/01]
40. Deeks JJ, Higgins JPT, Altman DG. (editors). Chapter 9: Analysing data and undertaking meta-analyses. In: Higgins JPT, Green S, eds. Cochrane handbook for systematic reviews of interventions version 510 [updated March 2011]: The Cochrane Collaboration, 2011.
41. Sterne JAC, Egger M, Moher D. (editors). Chapter 10: Addressing reporting biases. In: Higgins JPT, Green S, eds. Cochrane handbook for systematic reviews of intervention version 510 [updated March 2011]: The Cochrane Collaboration, 2011.
42. SPSS Inc. PASW Statistics for Windows, Version 18.0. Chicago: SPSS Inc. (2009). [published Online First: 18.0]
43. Shakiba S, Shakiba B, Irani S. Unpublished abstracts can be invaluable. *Can Urol Assoc J* 2014;8(1-2):E60. doi: 10.5489/cuaj.1534
44. Mody R, Li S, Dover DC, et al. Twenty-five-year follow-up among survivors of childhood acute lymphoblastic leukemia: a report from the Childhood Cancer Survivor Study. *Blood* 2008;111(12):5515-23. doi: 10.1182/blood-2007-10-117150 [published Online First: 2008/03/13]

For peer review only

For peer review only - http://bmjopen.bmj.com/site/about/guidelines.xhtml

Legends for tables and figures:

Supplementary File 1: PRISMA-P checklist. This checklist includes recommended items to address in a systematic review protocol and where are they reported in this protocol.

Supplementary File 2: Search Strategy for MEDLINE

Figure 1: Flow Diagram of Article Screening Process

For peer review only

For peer review only - http://bmjopen.bmj.com/site/about/guidelines.xhtml

Figure 1: Flow Diagram of Article Screening Process

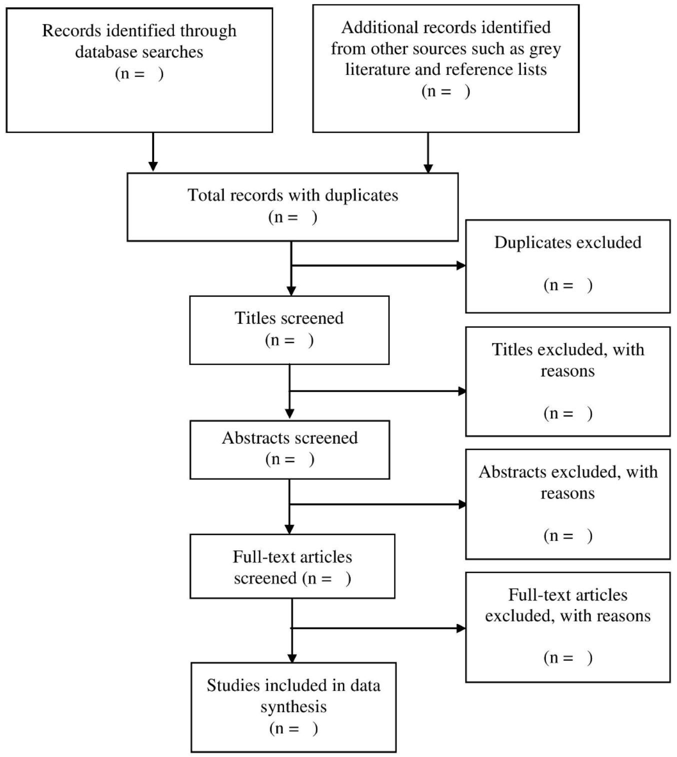

215x279mm (300 x 300 DPI)

Overweight and obesity management strategies in survivors of pediatric Acute Lymphoblastic Leukemia: A systematic review protocol

Salma Ladhani[1,2*], Brianna Empringham[1,2*], Kuan-Wen Wang[1,2,3], Carol Portwine[1,4], Laura Banfield[5], Russell J. de Souza[3,6], Lehana Thabane[3,6-9], M. Constantine Samaan[1,2,3,6#]
***These authors contributed equally to this work**

Salma Ladhani: salmaladhani@gmail.com
Brianna Empringham: brianna.empringham@medportal.ca
Kuan-Wen Wang: wangkw2@mcmaster.ca
Carol Portwine: portwc@mcmaster.ca
Laura Banfield: banfie@mcmaster.ca
Russell J. de Souza: rdesouz@mcmaster.ca
Lehana Thabane: thabanl@mcmaster.ca
M. Constantine Samaan: samaanc@mcmaster.ca

Authors' affiliations:
[1]Department of Pediatrics, McMaster University, Hamilton, Ontario, Canada
[2]Division of Pediatric Endocrinology, McMaster Children's Hospital, Hamilton, Ontario, Canada
[3]Medical Sciences Graduate Program, McMaster University, Hamilton, Ontario, Canada
[4]Division of Pediatric Hematology/Oncology, McMaster Children's Hospital, Hamilton, Ontario, Canada
[5]Health Sciences Library, McMaster University, Hamilton, Ontario, Canada
[6]Department of Health Research Methods, Evidence and Impact, McMaster University, Hamilton, Ontario, Canada
[7]Department of Anesthesia, McMaster University, Hamilton, Ontario, Canada
[8]Centre for Evaluation of Medicines, St. Joseph's Health Care, Hamilton, Ontario, Canada
[9]Biostatistics Unit, St Joseph's Healthcare, Hamilton, Ontario, Canada

# Corresponding Author:
Dr. M. Constantine Samaan
Department of Pediatrics,
McMaster University,
Division of Pediatric Endocrinology
McMaster Children's Hospital,
1280 Main Street West, HSC-3A57,

BioMed Central
The Open Access Publisher

For peer review only

For peer review only - http://bmjopen.bmj.com/site/about/guidelines.xhtml

Hamilton, Ontario L8S 4K1
Tel: 001-905-521-2100, ext. 75926,
Fax: 001-905-3087548
E-mail:samaanc@mcmaster.ca

For peer review only

BioMed Central The Open Access Publisher

Supplementary File 1: PRISMA-P Checklist

Section/topic	#	Checklist item	Information reported		Line number(s)
			Yes	No	
**ADMINISTRATIVE INFORMATION**					
**Title**					
Identification	1a	Identify the report as a protocol of a systematic review	☒	☐	2
Update	1b	If the protocol is for an update of a previous systematic review, identify as such	☐	☒	N/A
**Registration**	2	If registered, provide the name of the registry (e.g., PROSPERO) and registration number in the Abstract	☒	☐	68
**Authors**					
Contact	3a	Provide name, institutional affiliation, and e-mail address of all protocol authors; provide physical mailing address of corresponding author	☒	☐	4-40
Contributions	3b	Describe contributions of protocol authors and identify the guarantor of the review	☒	☐	311-316
**Amendments**	4	If the protocol represents an amendment of a previously completed or published protocol, identify as such and list changes; otherwise, state plan for documenting important protocol amendments	☒	☐	134-135
**Support**					
Sources	5a	Indicate sources of financial or other support for the review	☒	☐	302-305
Sponsor	5b	Provide name for the review funder and/or sponsor	☒	☐	302-305
Role of sponsor/funder	5c	Describe roles of funder(s), sponsor(s), and/or institution(s), if any, in developing the protocol	☒	☐	305
**INTRODUCTION**					
**Rationale**	6	Describe the rationale for the review in the context of what is already known	☒	☐	93-122
**Objectives**	7	Provide an explicit statement of the question(s) the review will address with reference to participants, interventions, comparators, and outcomes (PICO)	☒	☐	123-130
**METHODS**					
**Eligibility criteria**	8	Specify the study characteristics (e.g., PICO, study design, setting, time frame) and report characteristics (e.g., years considered, language, publication status) to be used as criteria for eligibility for the review	☒	☐	138-159
**Information sources**	9	Describe all intended information sources (e.g., electronic databases, contact with study authors, trial registers, or other grey literature sources) with planned dates of coverage	☒	☐	177-182
**Search strategy**	10	Present draft of search strategy to be used for at least one electronic database, including planned limits, such that it could be repeated	☒	☐	Supplementary File 2
**STUDY RECORDS**					
Data management	11a	Describe the mechanism(s) that will be used to manage records and data throughout the review	☒	☐	184-185

BioMed Central
The Open Access Publisher

For peer review only

Section/topic	#	Checklist item	Information reported		Line number(s)
			Yes	No	
Selection process	11b	State the process that will be used for selecting studies (e.g., two independent reviewers) through each phase of the review (i.e., screening, eligibility, and inclusion in meta-analysis)	☒	☐	187-194 235-237
Data collection process	11c	Describe planned method of extracting data from reports (e.g., piloting forms, done independently, in duplicate), any processes for obtaining and confirming data from investigators	☒	☐	196-198 207-208
**Data items**	12	List and define all variables for which data will be sought (e.g., PICO items, funding sources), any pre-planned data assumptions and simplifications	☒	☐	197-204
**Outcomes and prioritization**	13	List and define all outcomes for which data will be sought, including prioritization of main and additional outcomes, with rationale	☒	☐	161-175
**Risk of bias in individual studies**	14	Describe anticipated methods for assessing risk of bias of individual studies, including whether this will be done at the outcome or study level, or both; state how this information will be used in data synthesis	☒	☐	209-226
**DATA**					
**Synthesis**	15a	Describe criteria under which study data will be quantitatively synthesized	☒	☐	234-237
	15b	If data are appropriate for quantitative synthesis, describe planned summary measures, methods of handling data, and methods of combining data from studies, including any planned exploration of consistency (e.g., I^2, Kendall's tau)	☒	☐	238-246
	15c	Describe any proposed additional analyses (e.g., sensitivity or subgroup analyses, meta-regression)	☒	☐	247-251
	15d	If quantitative synthesis is not appropriate, describe the type of summary planned	☒	☐	237-238 255-256
**Meta-bias(es)**	16	Specify any planned assessment of meta-bias(es) (e.g., publication bias across studies, selective reporting within studies)	☒	☐	251-255
**Confidence in cumulative evidence**	17	Describe how the strength of the body of evidence will be assessed (e.g., GRADE)	☒	☐	227-233

This checklist has been adapted for use with systematic review protocol submissions to BioMed Central journals from Table 3 in Moher D et al**:** Preferred reporting items for systematic review and meta-analysis protocols (PRISMA-P) 2015 statement. *Systematic Reviews* 2015 **4**:1

An Editorial from the Editors-in-Chief of *Systematic Reviews* details why this checklist was adapted - **Moher D, Stewart L & Shekelle P:** Implementing PRISMA-P: recommendations for prospective authors. *Systematic Reviews* 2016 **5**:15

BioMed Central
The Open Access Publisher

For peer review only

Overweight and obesity management strategies in survivors of pediatric Acute Lymphoblastic Leukemia: A systematic review protocol

Salma Ladhani[1,2*], Brianna Empringham[1,2*], Kuan-Wen Wang[1,2,3], Carol Portwine[1,4], Laura Banfield[5], Russell J. de Souza[3,6], Lehana Thabane[3,6-9], M. Constantine Samaan[1,2,3,6#]

***These authors contributed equally to this work**

Salma Ladhani: salmaladhani@gmail.com

Brianna Empringham: brianna.empringham@medportal.ca

Kuan-Wen Wang: wangkw2@mcmaster.ca

Carol Portwine: portwc@mcmaster.ca

Laura Banfield: banfie@mcmaster.ca

Russell J. de Souza: rdesouz@mcmaster.ca

Lehana Thabane: thabanl@mcmaster.ca

M. Constantine Samaan: samaanc@mcmaster.ca

Authors' affiliations:

[1]Department of Pediatrics, McMaster University, Hamilton, Ontario, Canada

[2]Division of Pediatric Endocrinology, McMaster Children's Hospital, Hamilton, Ontario, Canada

[3]Medical Sciences Graduate Program, McMaster University, Hamilton, Ontario, Canada

[4]Division of Pediatric Hematology/Oncology, McMaster Children's Hospital, Hamilton, Ontario, Canada

[5]Health Sciences Library, McMaster University, Hamilton, Ontario, Canada

For peer review only

[6]Department of Health Research Methods, Evidence and Impact, McMaster University, Hamilton, Ontario, Canada

[7]Department of Anesthesia, McMaster University, Hamilton, Ontario, Canada

[8]Centre for Evaluation of Medicines, St. Joseph's Health Care, Hamilton, Ontario, Canada

[9]Biostatistics Unit, St Joseph's Healthcare, Hamilton, Ontario, Canada

# Corresponding Author:

Dr. M. Constantine Samaan

Department of Pediatrics,

McMaster University,

Division of Pediatric Endocrinology

McMaster Children's Hospital,

1280 Main Street West, HSC-3A57,

Hamilton, Ontario L8S 4K1

Tel: 001-905-521-2100, ext. 75926,

Fax: 001-905-3087548

E-mail:samaanc@mcmaster.ca

For peer review only

Supplementary File 2: Search Strategy for MEDLINE

1. exp Precursor Cell Lymphoblastic Leukemia-Lymphoma/

2. lymphoblastic leuk?emia*.mp.

3. lymphoblastic lymphoma*.mp.

4. lymphocytic leuk?emia*.mp.

5. lymphoid leuk?emia*.mp.

6. acute t-cell leuk?emia*.mp.

7. lymphatic leuk?emia*.mp.

8. t-lymphocytic leuk?emia*.mp.

9. precursor t cell lymphoblastic leuk?emia*.mp.

10. t cell leuk?emia*.mp.

11. t lymphocytic leuk?emia*.mp.

12. or/1-11

13. exp Overweight/

14. obes*.mp.

15. overweight.mp.

16. over weight.mp.

17. or/13-16

18. Life Change Events/

19. Life Style/

20. lifestyle*.mp.

21. life style*.mp.

22. exp Diet/

23. diet*.mp.

24. exp Nutrition Therapy/

25. nutrition*.mp.

26. behavio?r*.mp.

27. exp Exercise Therapy/

28. kinesiotherap*.mp.

29. physical* activ*.mp.

30. exp Exercise/

31. exercis*.mp.

32. ((strength or weight or resistance) adj1 train*).mp.

33. walk*.mp.

34. jog*.mp.

35. run*.mp.

36. swim*.mp.

37. exp Bariatrics/

38. bariatric*.mp.

39. gastrojejunostom*.mp.

40. gastric bypass*.mp.

41. stomach bypass*.mp.

42. jejunoileal bypass*.mp.

43. lipectom*.mp.

44. gastroplast*.mp.

45. stomach stapl*.mp.

46. exp Anti-Obesity Agents/

47. (drug or drugs).mp.

48. pharma*.mp.

49. Weight Reduction Programs/

50. ((weight reduc* or weight lo?s*) adj5 (strateg* or surger* or program* or system* or supplement*)).mp.

51. or/13-50

52. 12 and 17 and 51

peer review only

BMJ Open

Overweight and obesity management strategies in survivors of pediatric Acute Lymphoblastic Leukemia: A systematic review protocol

Journal:	*BMJ Open*
Manuscript ID	bmjopen-2018-022530.R1
Article Type:	Protocol
Date Submitted by the Author:	29-Mar-2018

Complete List of Authors:	Ladhani, Salma; McMaster University Department of Pediatrics,<br>Empringham, Brianna; McMaster University, Department of Pediatrics; McMaster Children's Hospital, Division of Pediatric Endocrinology<br>Wang, Kuan-Wen; McMaster University, Department of Paediatrics, Division of Pediatric Endocrinology<br>Portwine, Carol; McMaster University, Department of Pediatrics; McMaster Children's Hospital, Division of Pediatric Hematology/Oncology<br>Banfield, Laura; McMaster University, Health Sciences Library<br>de Souza, Russell; McMaster University, Department of Clinical Epidemiology & Biostatistics<br>Thabane, Lehana ; McMaster University, Clinical Epidemiology & Biostatistics<br>Samaan, M. Constantine; McMaster University, Pediatrics

**Primary Subject Heading**:	Diabetes and endocrinology
Secondary Subject Heading:	Oncology
Keywords:	Cancer survivorship, Obesity, Cancer aftereffects, Lifestyle interventions, Acute Lymphoblastic Leukemia

SCHOLARONE™
Manuscripts

Overweight and obesity management strategies in survivors of pediatric Acute Lymphoblastic Leukemia: A systematic review protocol

Salma Ladhani[1,2*], Brianna Empringham[1,2*], Kuan-Wen Wang[1,2,3], Carol Portwine[1,4], Laura Banfield[5], Russell J. de Souza[3,6], Lehana Thabane[3,6-9], M. Constantine Samaan[1,2,3,6#]

*These authors contributed equally to this work

Salma Ladhani: salmaladhani@gmail.com

Brianna Empringham: brianna.empringham@medportal.ca

Kuan-Wen Wang: wangkw2@mcmaster.ca

Carol Portwine: portwc@mcmaster.ca

Laura Banfield: banfie@mcmaster.ca

Russell J. de Souza: rdesouz@mcmaster.ca

Lehana Thabane: thabanl@mcmaster.ca

M. Constantine Samaan: samaanc@mcmaster.ca

Authors' affiliations:

[1]Department of Pediatrics, McMaster University, Hamilton, Ontario, Canada

[2]Division of Pediatric Endocrinology, McMaster Children's Hospital, Hamilton, Ontario, Canada

[3]Medical Sciences Graduate Program, McMaster University, Hamilton, Ontario, Canada

[4]Division of Pediatric Hematology/Oncology, McMaster Children's Hospital, Hamilton, Ontario, Canada

[5]Health Sciences Library, McMaster University, Hamilton, Ontario, Canada

For peer review only

[6]Department of Health Research Methods, Evidence and Impact, McMaster University, Hamilton, Ontario, Canada
[7]Department of Anesthesia, McMaster University, Hamilton, Ontario, Canada
[8]Centre for Evaluation of Medicines, St. Joseph's Health Care, Hamilton, Ontario, Canada
[9]Biostatistics Unit, St Joseph's Healthcare, Hamilton, Ontario, Canada

# Corresponding Author:
Dr. M. Constantine Samaan
Department of Pediatrics,
McMaster University,
Division of Pediatric Endocrinology
McMaster Children's Hospital,
1280 Main Street West, HSC-3A57,
Hamilton, Ontario L8S 4K1
Tel: 001-905-521-2100, ext. 75926,
Fax: 001-905-3087548
E-mail:samaanc@mcmaster.ca

Word count: 2003

For peer review only

Abstract

Introduction: Acute lymphoblastic leukemia is the most common pediatric cancer. Survivors of childhood acute lymphoblastic leukemia (SALL) are at risk of obesity and related cardiometabolic diseases including type 2 diabetes, hypertension, stroke and cardiovascular events, it is important to address obesity in this population to improve their survival and quality of life. In this systematic review, we aim to assess the effectiveness of current interventions to manage overweight and obesity in SALL.

Methods and analysis: We will search the following databases for primary studies: CINAHL, SPORTDiscus, EMBASE, MEDLINE, PsycINFO, Cochrane Central Register of Controlled Trials, Cochrane Database of Systematic Review, and Database of Abstracts of Reviews of Effect. In addition, unpublished primary studies will be searched in ClinicalTrials.gov and ProQuest Dissertations and Theses A&I. Reviewers will perform title, abstract, full-text screening, data abstraction, and risk of bias assessment independently with a third reviewer to be consulted to resolve disagreements. The overall quality of evidence will be determined using the Grading of Recommendations, Assessment, Development, and Evaluation (GRADE) criteria for each outcome. Meta-analysis will be done if two studies deploying similar interventions with similar population and study design are identified.

Discussion: We will summarize current understanding of interventions that target overweight and obesity in SALL, including lifestyle, pharmacotherapy and bariatric surgery. The results will provide critical insights into determining the best strategies to manage obesity in SALL and identify gaps in current knowledge.

Ethics and dissemination: As individual patient data will not be included, we do not require ethics approval. This review will be published in a peer-reviewed journal.

For peer review only

For peer review only - http://bmjopen.bmj.com/site/about/guidelines.xhtml

Systematic review registration: PROSPERO CRD42016051031

Keywords: acute lymphoblastic leukemia, pediatrics, survivorship, obesity, systematic review, lifestyle, pharmacotherapy, bariatric surgery

Strengths and Limitations

- This study will present a summary of the most recent research of obesity interventions in SALL, while also discerning gaps in current research in order to provide direction for design of future interventions.
- We plan to use the GRADE tool to analyse the overall quality of evidence, which provides an overview of the strength of the evidence.
- We expect high heterogeneity across studies, which may place challenges on performing a meta-analysis.

For peer review only

BACKGROUND

Childhood leukemia is the most common pediatric cancer,[1] with the most common subtype being acute lymphoblastic leukemia (ALL). An estimated 2670 children were diagnosed with ALL in the USA in 2014, representing 26% of all childhood cancer diagnoses. There are close to 380,000 survivors of childhood cancer living in the USA, the majority of whom are over twenty years of age, and are leukemia survivors.[1]

Remarkable advances in the characterization and treatment of ALL have improved survival rates, with 90% of children diagnosed with ALL surviving beyond 5 years today.[2 3] However, survivors of childhood ALL (SALL) are at risk of diseases that influence their long-term outcomes.

One of the common morbidities seen in this population is obesity.[4 5] It has been reported that the 38% of SALL are overweight or obese, compared to 31% of the general pediatric population.[4] Central obesity when associated with dysglycemia, hypertension and dyslipidemia is referred to as the metabolic syndrome, which is a risk factor for cardiovascular disease and type 2 diabetes.[4 6] The prevalence of metabolic syndrome in SALL is slightly higher than that in the general population.[7]

Despite having only modestly higher obesity rates compared to non-cancer controls, SALL have a disproportionately increased risk of insulin resistance and type 2 diabetes, as well as cardiovascular disease, stroke, and hypertension at a relatively young age.[8 9]

Several risk factors drive the risk of overweight/obesity and adverse cardiometabolic outcomes in survivors, including treatment with cranial irradiation and corticosteroids,[4 10-13] young age at diagnosis (0-4 years),[11 14-16] being overweight or obese at diagnosis,[15 16] and female sex.[11 16-18]

For peer review only - http://bmjopen.bmj.com/site/about/guidelines.xhtml

Addressing obesity in SALL is imperative, for the development of obesity in children can lead to obesity in adulthood and this raises the probability of adverse cardiometabolic outcomes.[19-21]

Understanding the risk factors of overweight and obesity in SALL will enable the development of targeted interventions to improve long-term health and outcomes by lowering the risk of obesity and its comorbidities. This systematic review aims to assess current interventions to manage overweight and obesity in SALL, and to evaluate their impact on adiposity and associated metabolic comorbidities.

RESEARCH QUESTION: In SALL, are lifestyle interventions, pharmacotherapy or bariatric surgery effective in treating overweight and obesity?

OBJECTIVES

1) To determine the effectiveness of existing interventions to manage overweight and obesity in SALL.
2) If feasible, to perform a meta-analysis of included studies and calculate a precise estimate of the effectiveness management strategies in SALL
3) Provide future research directions by identifying gaps in current evidence

METHODS

The methodology for this protocol has been established and reported based on the Preferred Reporting Items for Systematic Review and Meta-Analysis Protocols (PRISMA-P) guideline (Supplementary File 1).[22 23] When changes to the protocol are necessary, we will document the details for the rationale and details of the change in the reported systematic review.

Patient and public involvement

Patients and the public were not involved in the design of this review protocol.

For peer review only - http://bmjopen.bmj.com/site/about/guidelines.xhtml

Eligibility criteria

The studies sought will include SALL diagnosed before the age of 18 years. All subtypes of ALL will be eligible including T and B cell leukemias. Studies that involve all interventions targeting overweight and obesity in this population will be included. If there are studies that include other types of childhood cancers, we will check for subgroup analysis by cancer type and extract the data for the SALL population exclusively. If this information is not reported, we will contact the study Principal Investigators to obtain the data.

We will include interventions encompassing lifestyle, pharmacotherapy and bariatric surgery. Lifestyle interventions are defined as treatments that mainly involve exercise and dietary modifications. Pharmacotherapy involves the use of medications for the purpose of weight management. Bariatric surgery is defined as any surgical intervention with the primary goal of treating obesity. All current bariatric surgery procedures will be incorporated into this review including gastric bypass, biliopancreatic diversion with duodenal switch, sleeve gastrectomy, and gastric banding.[24]

The eligible study designs include randomized control trials (RCTs), quasi RCTs, cohort studies, case-control studies, controlled or uncontrolled studies with before-and-after comparisons, cross-sectional studies and pilot studies. Review articles, including systematic reviews, will be searched for relevant references if applicable to the research question. Conference proceedings, presentations, abstracts and editorials will be searched for relevant references as well. We will restrict the language of publication to English. Studies published up to August 2017 will be included, and updated searches will be done to ensure the inclusion of new papers prior to the completion of the review.

For peer review only

Outcome measures

Primary outcome

The primary outcome in this review involves changes in body mass index (BMI) z-score before and after the intervention.

Secondary outcomes

The secondary outcomes will include changes in adiposity measures including waist circumference, hip circumference, waist-to-hip ratio, waist-to-height ratio, and body fat percentage when reported. In addition, we will evaluate changes in insulin sensitivity, blood pressure, non-alcoholic fatty liver disease status, dyslipidemia (including cholesterol, triglycerides, high-density lipoprotein, and low-density lipoprotein), and obstructive sleep apnea.

We will also document any adverse effects of the interventions if reported. For lifestyle interventions, these include injuries, back pain, and angina.[25 26] For example, for pharmacotherapy these encompass headaches and insomnia among others.[27] For bariatric surgery, these include perioperative outcomes and surgical complications.[28] Other adverse effects will be described if reported.

Search Strategy

When designing the search strategy, we will consult a Senior Health Sciences Librarian with expertise in designing search strategies for systematic reviews. A sample MEDLINE search strategy is reported in Supplementary File 2. Searches will be conducted in CINAHL, SPORTDiscus, EMBASE, MEDLINE, PsycINFO, Cochrane Central Register of Controlled Trials, Cochrane Database of Systematic Review, and Database of Abstracts of Reviews of Effect. To identify grey literature, we will search ClinicalTrials.gov and ProQuest Dissertations and Theses A&I.

For peer review only - http://bmjopen.bmj.com/site/about/guidelines.xhtml

Data Management

We will use EndNote X7[29] to remove duplications from the search results, and references will be exported to an excel file.

Data Screening

We will remove duplicate references, and perform initial screening by two teams each composed of two reviewers independently for titles and abstracts. Differences in the included titles and abstracts will be discussed between the two reviewers in each team. A third reviewer will address unresolved conflicts at each step of the review process. The reviewers will screen all abstracts, and if there is uncertainty in the eligibility of the abstracts, they will be included. For all abstracts deemed eligible for inclusion, the full text papers will be retrieved and will undergo a full review by the two reviewers in each team independently. A flow diagram will be included to track the review process (Figure 1).

Data Abstraction

Pertinent data to the study question will be abstracted using a data abstraction form specifically designed for this project. This form incorporates study details including authors, title, study date, country, study design, inclusion/exclusion criteria, journal of publication, and funding details. It also incorporates participant details including age, sex, ethnicity, age at ALL diagnosis, and time from diagnosis to intervention if available.

Other data extracted include treatment types with radiotherapy field (cranial, craniospinal, total body irradiation), radiation type (fractionated versus non-fractionated) and radiation dose. We will also include chemotherapy protocols, name of medications, dose, and duration of treatment. In addition, medical as well as metabolic comorbidities will be abstracted.

The targeted obesity intervention details will be abstracted including study design,

For peer review only - http://bmjopen.bmj.com/site/about/guidelines.xhtml

intervention details, duration and follow-up periods, and any reported side effects. Details of the statistics performed and results will be abstracted. In order to retrieve incomplete data, we will attempt to contact the Principal Investigators of the published work. Outcomes will be summarized, along with the overall conclusions and any confounding factors.

Risk of Bias & Quality Assessment

The Risk of Bias Assessment Tool created by the Cochrane Collaboration will be used to evaluate the risk of bias for RCTs.[30] The studies will be rated as having a high, low or unclear risk of bias based on the validity of sequence generation, allocation concealment, blinding, data completeness, selective reporting and other sources of bias.

Non-randomized trials, including cohort studies, will be assessed using the Risk of Bias in Non-Randomized Studies- of Interventions (ROBINS-I) tool.[31] This tool considers confounding variables and selection bias at the pre-intervention stage. The following confounding factors will be considered when using this tool: Age at time of enrolment in the intervention, age at diagnosis, pubertal stage, sex, baseline body composition, treatments received for ALL, years since end of therapy, and comorbidities such as metabolic syndrome and hormonal deficiencies. At the intervention stage, misclassification of intervention group will be evaluated. Lastly, deviation from intended interventions, missing data, standardized methods of outcome measures, and selective reporting will be assessed at the post-intervention stage.

In uncontrolled before and after studies, the risk of bias will be assessed using the University of Alberta Evidence-based Practice Center (UAEPC) checklist.[32] This checklist looks at patient enrollment, incomplete data and standardized approach to outcomes to determine the risk of bias.

For peer review only

For peer review only - http://bmjopen.bmj.com/site/about/guidelines.xhtml

The overall quality of the body of evidence will be evaluated with the Grading of Recommendations, Assessment, Development, and Evaluation (GRADE) approach.[33] We will consider the quality of the evidence and the magnitude of the effect to determine the overall strength of the meta-analysis findings if it is feasible to do so. The determinants of quality include risk of bias, consistency of results across studies, indirectness/heterogeneity, imprecision and publication bias. The overall strength of evidence will be classified as high, moderate, low or very low for each outcome measure.

Data Analysis

We will include the summary and details of each relevant study in our analysis. If two studies on similar interventions with similar population and study design are identified, a meat-analysis will be conducted. Otherwise, a narrative summary with a detailed table of findings will be presented. Categorical outcomes will be reported as odds ratios, and continuous variables will be reported as mean differences with 95% confidence interval.

We will perform meta-analysis with a random effects model if more than ten studies can be included for meta-analysis using Review Manager software.[34] We are expecting high heterogeneity across studies, and this random effects model is the preferred method in this case.[35] Otherwise, we will present results from both random and fixed effects models.[36]

Heterogeneity across studies will be evaluated with inconsistency index (I^2) and p-values from chi-square test for homogeneity, with I^2 >75% and a p-value of <0.10 representing considerable heterogeneity.[37]

If appropriate, we will perform subgroup meta-analysis by sex, as female SALL has been reported to have higher risk of obesity than male SALL and may respond to overweight and obesity management strategies differently.[11 16-18] We will determine publication bias by creating

For peer review only - http://bmjopen.bmj.com/site/about/guidelines.xhtml

a funnel plot if ten or more studies are included.[38] We will use Egger's test, performed with PASW version 18 statistical package,[39] and visual inspection to determine the plot asymmetry. Alternatively, we will estimate publication bias by considering the number of relevant conference abstracts without published articles. If a meta-analysis is not appropriate, a summary table with narrative description will be reported.

DISCUSSION

Improvements in treatments and supportive care have led to an increased survival in children diagnosed with ALL. However, comorbidities in SALL are high and affect quality of life and long-term outcomes. The increased rates of obesity in this population represent a potential area of need for intervention to help improve cardiometabolic outcomes in SALL. Around 70% of survivors of childhood cancer have at least one chronic health issue with limitations of activity and lower quality of life.[40] It is vitally important to understand if effective overweight and obesity interventions are available for SALL, as treatment is associated with high cure rates. As this population gets older, survivors are forced to live longer with the burden of their cancer history. This study will summarize the current scientific understanding of interventions that target overweight and obesity in SALL, and will identify gaps in current research, and guide future study design to improve outcomes.

Ethics and dissemination: We will not include individual patient data in this review. Therefore, ethics approval is not required, and there are no further ethical and safety considerations. The dissemination plan for this review is to be published in a peer-reviewed journal.

For peer review only

ABBREVIATIONS

ALL: Acute Lymphoblastic Leukemia; SALL: Survivors of childhood Acute Lymphoblastic Leukemia; PRISMA-P: Preferred Reporting Items for Systematic Review and Meta-Analysis-Protocols; RCT: Randomized Control Trial; BMI: Body Mass Index; MEDLINE: Medical Literature Analysis and Retrieval System; EMBASE: Excerpta Medica Database; CINAHL: Cumulative Index to Nursing and Allied Health Literature; GRADE: Grading of Recommendations, Assessment, Development and Evaluation; ROBINS-I: Risk of Bias in Non-Randomized Studies – of Interventions; UAEPC: University of Alberta Evidence-based Practice Center.

For peer review only

For peer review only - http://bmjopen.bmj.com/site/about/guidelines.xhtml

DECLARATIONS

Ethics approval and consent to participate: Not applicable

Consent for publication: Not applicable

Availability of data and materials: Not applicable

Competing interests: The authors declare no competing interests.

Funding: M.C.S. was funded by the Pediatric Oncology Group of Ontario (POGO) Research Unit, and the Hamilton Health Sciences and Foundation, K.W.W. was funded by the Ontario Graduate Scholarship Program, and the Canadian Institutes of Health Research (CIHR) Canada Graduate Scholarship-Masters. Design of the systematic review is independent of the funding agencies.

Authors' contributions: MCS is the guarantor. Research question was defined by SL, BE, CP, RJdS, LT and MCS. SL, BE, KWW, CP, LB, RJdS, LT and MCS contributed to the development of search strategy and determination of the eligibility criteria. SL, BE, LB, KWW and MCS designed the data abstraction form. RJdS and LT provided methodological support for this review. SL, BE, KWW, CP and MCS wrote the first draft of the manuscript, and the final version was reviewed and approved by all authors.

For peer review only

For peer review only - http://bmjopen.bmj.com/site/about/guidelines.xhtml

References

1. Ward E, DeSantis C, Robbins A, et al. Childhood and adolescent cancer statistics, 2014. *CA: A Cancer Journal for Clinicians* 2014;64(2):83-103. doi: 10.3322/caac.21219

2. Pui CH, Evans WE. Treatment of acute lymphoblastic leukemia. *N Engl J Med* 2006;354(2):166-78. doi: 10.1056/NEJMra052603 [published Online First: 2006/01/13]

3. Pulte D, Gondos A, Brenner H. Trends in 5- and 10-year Survival After Diagnosis with Childhood Hematologic Malignancies in the United States, 1990–2004. *JNCI: Journal of the National Cancer Institute* 2008;100(18):1301-09. doi: 10.1093/jnci/djn276

4. Chow EJ, Pihoker C, Hunt K, et al. Obesity and hypertension among children after treatment for acute lymphoblastic leukemia. *Cancer* 2007;110(10):2313-20. doi: 10.1002/cncr.23050 [published Online First: 2007/09/28]

5. Withycombe JS, Smith LM, Meza JL, et al. Weight change during childhood acute lymphoblastic leukemia induction therapy predicts obesity: a report from the Children's Oncology Group. *Pediatr Blood Cancer* 2015;62(3):434-9. doi: 10.1002/pbc.25316 [published Online First: 2014/11/20]

6. Expert Panel on Detection, Evaluation, and Treatment of High Blood Cholesterol in Adults. Executive Summary of The Third Report of The National Cholesterol Education Program (NCEP) Expert Panel on Detection, Evaluation, And Treatment of High Blood Cholesterol In Adults (Adult Treatment Panel III). *JAMA* 2001;285(19):2486-97. [published Online First: 2001/05/23]

7. Kourti M, Tragiannidis A, Makedou A, et al. Metabolic syndrome in children and adolescents with acute lymphoblastic leukemia after the completion of chemotherapy. *J Pediatr Hematol Oncol* 2005;27(9):499-501. [published Online First: 2005/09/29]

8. Oeffinger KC. Are survivors of acute lymphoblastic leukemia (ALL) at increased risk of cardiovascular disease? *Pediatric Blood & Cancer* 2008;50(S2):462-67. doi: 10.1002/pbc.21410

9. Oeffinger KC, Adams-Huet B, Victor RG, et al. Insulin Resistance and Risk Factors for Cardiovascular Disease in Young Adult Survivors of Childhood Acute Lymphoblastic Leukemia. *Journal of Clinical Oncology* 2009;27(22):3698-704. doi: doi:10.1200/JCO.2008.19.7251

10. Garmey EG, Liu Q, Sklar CA, et al. Longitudinal changes in obesity and body mass index among adult survivors of childhood acute lymphoblastic leukemia: a report from the Childhood Cancer Survivor Study. *J Clin Oncol* 2008;26(28):4639-45. doi: 10.1200/JCO.2008.16.3527 [published Online First: 2008/10/01]

11. Oeffinger KC, Mertens AC, Sklar CA, et al. Obesity in Adult Survivors of Childhood Acute Lymphoblastic Leukemia: A Report from the Childhood Cancer Survivor Study. *Journal of Clinical Oncology* 2003;21(7):1359-65. doi: doi:10.1200/JCO.2003.06.131

12. Sklar CA, Mertens AC, Walter A, et al. Changes in body mass index and prevalence of overweight in survivors of childhood acute lymphoblastic leukemia: role of cranial irradiation. *Med Pediatr Oncol* 2000;35(2):91-5. [published Online First: 2000/08/05]

13. van Beek RD, de Muinck Keizer-Schrama SM, Hakvoort-Cammel FG, et al. No difference between prednisolone and dexamethasone treatment in bone mineral density and growth in long term survivors of childhood acute lymphoblastic leukemia. *Pediatr Blood Cancer* 2006;46(1):88-93. doi: 10.1002/pbc.20437 [published Online First: 2005/06/01]

14. Dalton VK, Rue M, Silverman LB, et al. Height and weight in children treated for acute lymphoblastic leukemia: relationship to CNS treatment. *J Clin Oncol* 2003;21(15):2953-60. doi: 10.1200/jco.2003.03.068 [published Online First: 2003/07/30]

15. Razzouk BI, Rose SR, Hongeng S, et al. Obesity in survivors of childhood acute lymphoblastic leukemia and lymphoma. *J Clin Oncol* 2007;25(10):1183-9. doi: 25/10/1183 [pii]

For peer review only

10.1200/JCO.2006.07.8709 [doi] [published Online First: 2007/04/03]
16. Withycombe JS, Post-White JE, Meza JL, et al. Weight Patterns in Children With Higher Risk ALL: A Report From the Children's Oncology Group (COG) for CCG 1961. *Pediatric blood & cancer* 2009;53(7):1249-54. doi: 10.1002/pbc.22237
17. Kohler JA, Moon RJ, Wright S, et al. Increased adiposity and altered adipocyte function in female survivors of childhood acute lymphoblastic leukaemia treated without cranial radiation. *Horm Res Paediatr* 2011;75(6):433-40. doi: 10.1159/000324412 [published Online First: 2011/04/06]
18. Odame I, Reilly JJ, Gibson BE, et al. Patterns of obesity in boys and girls after treatment for acute lymphoblastic leukaemia. *Archives of Disease in Childhood* 1994;71(2):147-49.
19. Bray GA. Predicting obesity in adults from childhood and adolescent weight. *Am J Clin Nutr* 2002;76(3):497-8. [published Online First: 2002/08/29]
20. Guo SS, Wu W, Chumlea WC, et al. Predicting overweight and obesity in adulthood from body mass index values in childhood and adolescence. *Am J Clin Nutr* 2002;76(3):653-8. [published Online First: 2002/08/29]
21. Simmonds M, Llewellyn A, Owen CG, et al. Predicting adult obesity from childhood obesity: a systematic review and meta-analysis. *Obes Rev* 2016;17(2):95-107. doi: 10.1111/obr.12334
22. Moher D, Shamseer L, Clarke M, et al. Preferred reporting items for systematic review and meta-analysis protocols (PRISMA-P) 2015 statement. *Systematic reviews* 2015;4:1. doi: 10.1186/2046-4053-4-1 [published Online First: 2015/01/03]
23. Moher D, Stewart L, Shekelle P. Implementing PRISMA-P: recommendations for prospective authors. *Systematic Reviews* 2016;5(1):15. doi: 10.1186/s13643-016-0191-y
24. Buchwald H, Oien DM. Metabolic/bariatric surgery Worldwide 2008. *Obes Surg* 2009;19(12):1605-11. doi: 10.1007/s11695-009-0014-5
25. Ried-Larsen M, Christensen R, Hansen KB, et al. Head-to-head comparison of intensive lifestyle intervention (U-TURN) versus conventional multifactorial care in patients with type 2 diabetes: protocol and rationale for an assessor-blinded, parallel group and randomised trial. *BMJ Open* 2015;5(12) doi: 10.1136/bmjopen-2015-009764
26. Yang Z, Scott CA, Mao C, et al. Resistance Exercise Versus Aerobic Exercise for Type 2 Diabetes: A Systematic Review and Meta-Analysis. *Sports Medicine* 2014;44(4):487-99. doi: 10.1007/s40279-013-0128-8
27. Kang JG, Park CY. Anti-Obesity Drugs: A Review about Their Effects and Safety. *Diabetes Metab J* 2012;36(1):13-25. doi: 10.4093/dmj.2012.36.1.13
28. Hopkins JC, Howes N, Chalmers K, et al. Outcome reporting in bariatric surgery: an in-depth analysis to inform the development of a core outcome set, the BARIACT Study. *Obesity Reviews* 2015;16(1):88-106. doi: 10.1111/obr.12240
29. EndNote [Computer program]. Version 7.7.1 [program]: Clarivate Analytics, 2016.
30. Higgins JPT, Altman DG, Sterne JAC. (editors). Chapter 8: Assessing risk of bias in included studies. In: Higgins JPT, Green S, eds. Cochrane handbook for systematic review of interventions Version 510 [updated March 2011]: The Cochrane Collaboration, 2011.
31. Sterne JA, Hernan MA, Reeves BC, et al. ROBINS-I: a tool for assessing risk of bias in non-randomised studies of interventions. *BMJ* 2016;355:i4919. doi: 10.1136/bmj.i4919 [published Online First: 2016/10/14]
32. Seida JC, Schouten JR, Mousavi SS, et al. 2. Methods. Comparative effectiveness of nonoperative and operative treatment for rotator cuff tears Rockville (MD): Agency for Healthcare Research and Quality (US), 2010.
33. Atkins D, Best D, Briss PA, et al. Grading quality of evidence and strength of recommendations. *BMJ* 2004;328(7454):1490. doi: 10.1136/bmj.328.7454.1490

For peer review only - http://bmjopen.bmj.com/site/about/guidelines.xhtml

34. Review Manager (RevMan) [Computer program]. Version 5.3. [program]. Copenhagen: The Nordic Cochrane Centre, The Cochrane Collaboration, 2014.
35. Villar J, Mackey ME, Carroli G, et al. Meta-analyses in systematic reviews of randomized controlled trials in perinatal medicine: comparison of fixed and random effects models. *Stat Med* 2001;20(23):3635-47.
36. Borenstein M, Hedges LV, Higgins JP, et al. A basic introduction to fixed-effect and random-effects models for meta-analysis. *Research synthesis methods* 2010;1(2):97-111. doi: 10.1002/jrsm.12 [published Online First: 2010/04/01]
37. Deeks JJ, Higgins JPT, Altman DG. (editors). Chapter 9: Analysing data and undertaking meta-analyses. In: Higgins JPT, Green S, eds. Cochrane handbook for systematic reviews of interventions version 510 [updated March 2011]: The Cochrane Collaboration, 2011.
38. Sterne JAC, Egger M, Moher D. (editors). Chapter 10: Addressing reporting biases. In: Higgins JPT, Green S, eds. Cochrane handbook for systematic reviews of intervention version 510 [updated March 2011]: The Cochrane Collaboration, 2011.
39. Alexander RW. President's Address: Common Mechanisms of Multiple Diseases: Why Vegetables and Exercise Are Good for You. *Trans Am Clin Climatol Assoc*;121:1-20.
40. Phillips SM, Padgett LS, Leisenring WM, et al. Survivors of childhood cancer in the United States: prevalence and burden of morbidity. *Cancer Epidemiol Biomarkers Prev* 2015;24(4):653-63. doi: 10.1158/1055-9965.epi-14-1418 [published Online First: 2015/04/04]

For peer review only

For peer review only - http://bmjopen.bmj.com/site/about/guidelines.xhtml

Legends for tables and figures:

Supplementary File 1: PRISMA-P checklist. This checklist includes recommended items to address in a systematic review protocol and where are they reported in this protocol.

Supplementary File 2: Search Strategy for MEDLINE

Figure 1: Flow Diagram of Article Screening Process

For peer review only

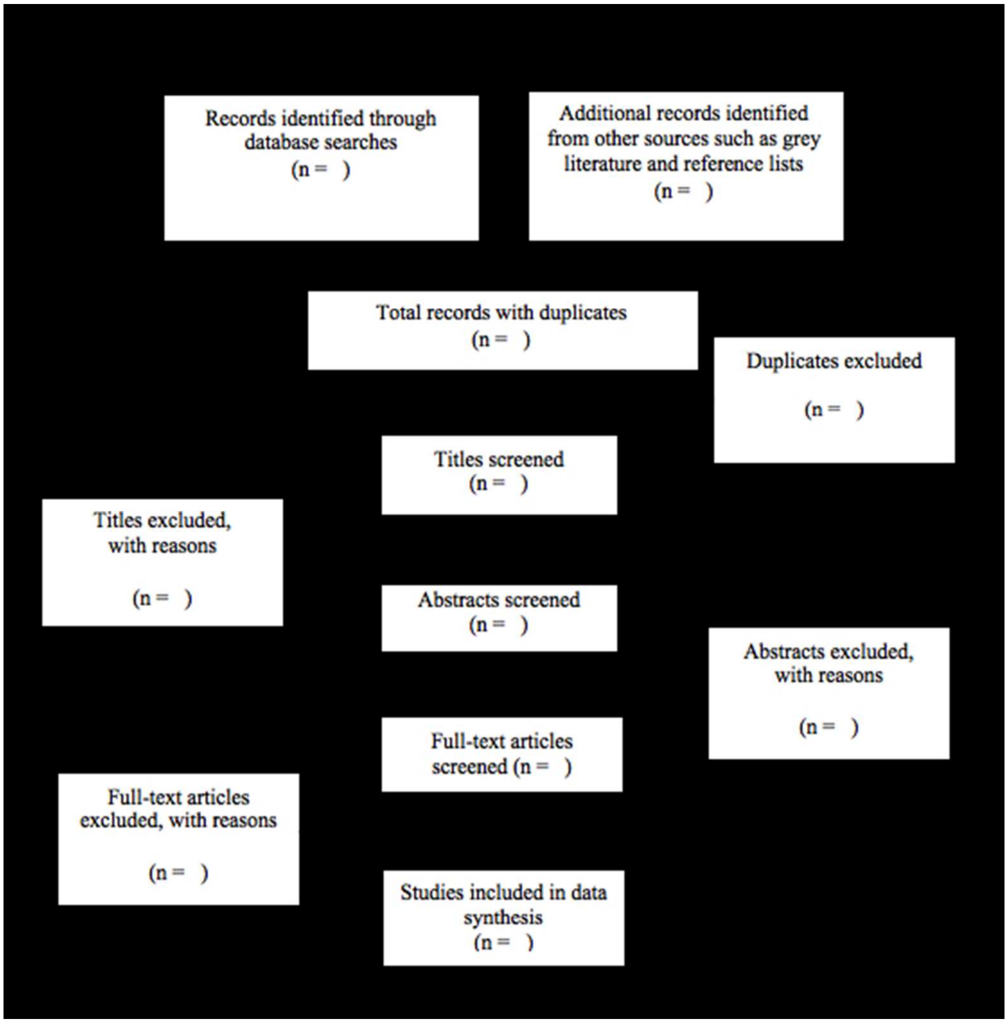

47x47mm (299 x 299 DPI)

Supplementary File 1: PRISMA-P Checklist

Section/topic	#	Checklist item	Information reported: Yes	Information reported: No	Line number(s)
**ADMINISTRATIVE INFORMATION**					
**Title**					
Identification	1a	Identify the report as a protocol of a systematic review	☒	☐	2
Update	1b	If the protocol is for an update of a previous systematic review, identify as such	☐	☒	N/A
**Registration**	2	If registered, provide the name of the registry (e.g., PROSPERO) and registration number in the Abstract	☒	☐	70
**Authors**					
Contact	3a	Provide name, institutional affiliation, and e-mail address of all protocol authors; provide physical mailing address of corresponding author	☒	☐	4-40
Contributions	3b	Describe contributions of protocol authors and identify the guarantor of the review	☒	☐	309-314
**Amendments**	4	If the protocol represents an amendment of a previously completed or published protocol, identify as such and list changes; otherwise, state plan for documenting important protocol amendments	☒	☐	136-137
**Support**					
Sources	5a	Indicate sources of financial or other support for the review	☒	☐	304-307
Sponsor	5b	Provide name for the review funder and/or sponsor	☒	☐	304-307
Role of sponsor/funder	5c	Describe roles of funder(s), sponsor(s), and/or institution(s), if any, in developing the protocol	☒	☐	307-308
**INTRODUCTION**					
**Rationale**	6	Describe the rationale for the review in the context of what is already known	☒	☐	93-124
**Objectives**	7	Provide an explicit statement of the question(s) the review will address with reference to participants, interventions, comparators, and outcomes (PICO)	☒	☐	125-132
**METHODS**					
**Eligibility criteria**	8	Specify the study characteristics (e.g., PICO, study design, setting, time frame) and report characteristics (e.g., years considered, language, publication status) to be used as criteria for eligibility for the review	☒	☐	139-160
**Information sources**	9	Describe all intended information sources (e.g., electronic databases, contact with study authors, trial registers, or other grey literature sources) with planned dates of coverage	☒	☐	177-184
**Search strategy**	10	Present draft of search strategy to be used for at least one electronic database, including planned limits, such that it could be repeated	☒	☐	Supplementary File 2
**STUDY RECORDS**					
Data management	11a	Describe the mechanism(s) that will be used to manage records and data throughout the review	☒	☐	185-187

For peer review only

BioMed Central The Open Access Publisher

Section/topic	#	Checklist item	Information reported Yes	Information reported No	Line number(s)
Selection process	11b	State the process that will be used for selecting studies (e.g., two independent reviewers) through each phase of the review (i.e., screening, eligibility, and inclusion in meta-analysis)	☒	☐	188-196
Data collection process	11c	Describe planned method of extracting data from reports (e.g., piloting forms, done independently, in duplicate), any processes for obtaining and confirming data from investigators	☒	☐	197-199 209-210
**Data items**	12	List and define all variables for which data will be sought (e.g., PICO items, funding sources), any pre-planned data assumptions and simplifications	☒	☐	199-206
**Outcomes and prioritization**	13	List and define all outcomes for which data will be sought, including prioritization of main and additional outcomes, with rationale	☒	☐	162-176
**Risk of bias in individual studies**	14	Describe anticipated methods for assessing risk of bias of individual studies, including whether this will be done at the outcome or study level, or both; state how this information will be used in data synthesis	☒	☐	212-229
**DATA**					
**Synthesis**	15a	Describe criteria under which study data will be quantitatively synthesized	☒	☐	237-240
	15b	If data are appropriate for quantitative synthesis, describe planned summary measures, methods of handling data, and methods of combining data from studies, including any planned exploration of consistency (e.g., I^2, Kendall's tau)	☒	☐	241-249
	15c	Describe any proposed additional analyses (e.g., sensitivity or subgroup analyses, meta-regression)	☒	☐	250-252
	15d	If quantitative synthesis is not appropriate, describe the type of summary planned	☒	☐	240-241 256-257
**Meta-bias(es)**	16	Specify any planned assessment of meta-bias(es) (e.g., publication bias across studies, selective reporting within studies)	☒	☐	252-256
**Confidence in cumulative evidence**	17	Describe how the strength of the body of evidence will be assessed (e.g., GRADE)	☒	☐	230-236

This checklist has been adapted for use with systematic review protocol submissions to BioMed Central journals from Table 3 in Moher D et al**:** Preferred reporting items for systematic review and meta-analysis protocols (PRISMA-P) 2015 statement. *Systematic Reviews* 2015 **4**:1

An Editorial from the Editors-in-Chief of *Systematic Reviews* details why this checklist was adapted - **Moher D, Stewart L & Shekelle P:** Implementing PRISMA-P: recommendations for prospective authors. *Systematic Reviews* 2016 **5**:15

BioMed Central The Open Access Publisher

Supplementary File 2: Search Strategy for MEDLINE

1. exp Precursor Cell Lymphoblastic Leukemia-Lymphoma/

2. lymphoblastic leuk?emia*.mp.

3. lymphoblastic lymphoma*.mp.

4. lymphocytic leuk?emia*.mp.

5. lymphoid leuk?emia*.mp.

6. acute t-cell leuk?emia*.mp.

7. lymphatic leuk?emia*.mp.

8. t-lymphocytic leuk?emia*.mp.

9. precursor t cell lymphoblastic leuk?emia*.mp.

10. t cell leuk?emia*.mp.

11. t lymphocytic leuk?emia*.mp.

12. or/1-11

13. exp Overweight/

14. obes*.mp.

15. overweight.mp.

16. over weight.mp.

17. or/13-16

18. Life Change Events/

19. Life Style/

20. lifestyle*.mp.

21. life style*.mp.

22. exp Diet/

23. diet*.mp.

24. exp Nutrition Therapy/

25. nutrition*.mp.

26. behavio?r*.mp.

27. exp Exercise Therapy/

28. kinesiotherap*.mp.

29. physical* activ*.mp.

30. exp Exercise/

31. exercis*.mp.

32. ((strength or weight or resistance) adj1 train*).mp.

33. walk*.mp.

34. jog*.mp.

35. run*.mp.

36. swim*.mp.

37. exp Bariatrics/

38. bariatric*.mp.

39. gastrojejunostom*.mp.

40. gastric bypass*.mp.

41. stomach bypass*.mp.

42. jejunoileal bypass*.mp.

43. lipectom*.mp.

44. gastroplast*.mp.

45. stomach stapl*.mp.

46. exp Anti-Obesity Agents/

47. (drug or drugs).mp.

48. pharma*.mp.

49. Weight Reduction Programs/

50. ((weight reduc* or weight lo?s*) adj5 (strateg* or surger* or program* or system* or supplement*)).mp.

51. or/13-50

52. 12 and 17 and 51

peer review only
