# Supplementary material for: Overweight and obesity management strategies in survivors of paediatric acute lymphoblastic leukaemia: a systematic review protocol
Source: BMJ Open. 2018 Jun 22;8(6):e022530. doi: 10.1136/bmjopen-2018-022530 (PMC6020978; doi:10.1136/bmjopen-2018-022530)
Supplement: Reviewer comments [file bmjopen-2018-022530.reviewer_comments.pdf]

PEER REVIEW HISTORY

BMJ Open publishes all reviews undertaken for accepted manuscripts. Reviewers are asked to complete a checklist review form (**http://bmjopen.bmj.com/site/about/resources/checklist.pdf**) and are provided with free text boxes to elaborate on their assessment. These free text comments are reproduced below.

ARTICLE DETAILS

**TITLE (PROVISIONAL)**	Overweight and obesity management strategies in survivors of pediatric Acute Lymphoblastic Leukemia: A systematic review protocol
**AUTHORS**	Ladhani, Salma; Empringham, Brianna; Wang, Kuan-Wen; Portwine, Carol; Banfield, Laura; de Souza, Russell; Thabane, Lehana; Samaan, M. Constantine

This article was not externally reviewed at BMJ Open. Protocols that have been independently assessed prior to submission to BMJ Open are usually fast-tracked to publication on the grounds that further substantial changes will not be possible. This independent assessment will usually be external, independent review for both a substantial grant award from a non-commercial or government funder and ethics approval.
